# PDHA1 hyperacetylation-mediated lactate overproduction promotes sepsis-induced acute kidney injury via Fis1 lactylation

**DOI:** 10.1038/s41419-023-05952-4

**Published:** 2023-07-21

**Authors:** Sheng An, Yi Yao, Hongbin Hu, Junjie Wu, Jiaxin Li, Lulan Li, Jie Wu, Maomao Sun, Zhiya Deng, Yaoyuan Zhang, Shenhai Gong, Qiaobing Huang, Zhongqing Chen, Zhenhua Zeng

**Affiliations:** 1 Department of Critical Care Medicine, Nanfang Hospital, Southern Medical University, Guangzhou, 510515 China; 2 Guangdong Provincial Key Laboratory of Cardiac Function and Microcirculation, Department of Pathophysiology, School of Basic Medical Sciences, Southern Medical University, Guangzhou, 510515 China; 3 School of Traditional Chinese Medicine, Southern Medical University, Guangzhou, 510515 China

**Keywords:** Proteomics, Acute kidney injury, Bacterial infection, Post-translational modifications, Mechanisms of disease

## Abstract

The increase of lactate is an independent risk factor for patients with sepsis-induced acute kidney injury (SAKI). However, whether elevated lactate directly promotes SAKI and its mechanism remain unclear. Here we revealed that downregulation of the deacetylase Sirtuin 3 (SIRT3) mediated the hyperacetylation and inactivation of pyruvate dehydrogenase E1 component subunit alpha (PDHA1), resulting in lactate overproduction in renal tubular epithelial cells. We then found that the incidence of SAKI and renal replacement therapy (RRT) in septic patients with blood lactate ≥ 4 mmol/L was increased significantly, compared with those in septic patients with blood lactate < 2 mmol/L. Further in vitro and in vivo experiments showed that additional lactate administration could directly promote SAKI. Mechanistically, lactate mediated the lactylation of mitochondrial fission 1 protein (Fis1) lysine 20 (Fis1 K20la). The increase in Fis1 K20la promoted excessive mitochondrial fission and subsequently induced ATP depletion, mitochondrial reactive oxygen species (mtROS) overproduction, and mitochondrial apoptosis. In contrast, PDHA1 activation with sodium dichloroacetate (DCA) or SIRT3 overexpression decreased lactate levels and Fis1 K20la, thereby alleviating SAKI. In conclusion, our results show that PDHA1 hyperacetylation and inactivation enhance lactate overproduction, which mediates Fis1 lactylation and exacerbates SAKI. Reducing lactate levels and Fis1 lactylation attenuate SAKI.

## Introduction

SAKI is one of the most common complications in patients with sepsis [1]. For patients in the intensive care unit (ICU), ~40–50% of AKI patients also have sepsis [2]. SAKI often results in poor prognosis and high mortality in these patients [3, 4]. Therefore, clarifying the pathogenesis of SAKI is critical for clinical treatment.

Pyruvate dehydrogenase (PDH) is the first rate-limiting enzyme that catalyzes the oxidative dehydrogenation of pyruvate to acetyl-CoA [5]. Subunit alpha of PDH (PDHA1) contains the PDH active site [6]. The activity of PDHA1 can be regulated by acetylation. PDHA1 acetylation reduces PDH activity, and deacetylation enhances PDH activity [6–9]. PDHA1 inactivation is believed to be involved in metabolic reprogramming and lactate overproduction in tumor [6]. Decreased PDH activity was observed in skeletal muscle cells and peripheral blood mononuclear cells during sepsis [10–13]. However, whether PDHA1 acetylation and activity are altered in SAKI remains unclear.

Sirtuin 3 (SIRT3) belongs to the sirtuins family, which contains seven members: SIRT1-7 [14]. SIRT3-5 are located in mitochondria and only SIRT3 is the strongest deacetylase located in mitochondria [15]. SIRT3 has been reported to deacetylate PDHA1 and reduce lactate generation in tumor and chronic renal fibrosis [6, 16, 17]. But whether PDHA1 acetylation is regulated by SIRT3 and thereby alters lactate production in SAKI should be further examined.

Lactate, which is an end product of glycolysis, is an independent predictor of poor prognosis in patients with sepsis [18]. In addition to being an energy source for mitochondrial respiration, lactate is involved in histone lysine lactylation [19]. Therefore, whether the increased in lactate during sepsis contributes to protein lactylation is of interest to researchers. Importantly, it is not clear whether nonhistone lactylation exists in the development of SAKI.

Mitochondria are highly dynamic organelles that maintain homeostasis through iterative fusion and fission. Disturbance in the balance between mitochondrial fission and fusion, such as excessive mitochondrial fission, alters energy metabolism, redox balance, and apoptosis and is a hallmark of various diseases [20, 21]. Mitochondrial fission 1 protein (Fis1), which is a mitochondrial outer membrane adaptor, interacts with the fission executor dynamin-related protein 1 (DRP1) to mediate mitochondrial fission [22–25]. There have been few reports about posttranslational modification of Fis1. A recent study showed that phosphorylated Fis1 mediated mitochondrial fragmentation [22]. However, it’s unclear whether Fis1 has other posttranslational modifications in SAKI development.

Here we examined the acetylation and lactylation changes in SAKI, and the results showed that PDHA1 was hyperacetylation and inactivation with concomitant lactate overproduction. The increase of lactate was associated with a poor prognosis for patients with SAKI and directly exacerbated SAKI in vitro and in vivo. Importantly, the lactate-mediated increase in Fis1 lysine 20 lactylation (Fis1 K20la) could promote excessive mitochondrial fission and subsequent dysfunction. Intriguingly, we evidenced that activating PDHA1 could decrease Fis1 K20la, and thus alleviate SAKI. These findings reveal that lactate-mediated lactylation, which is a novel posttranslational modification establishes a link between lactate and septic organ damage.

## Results

### The renal mitochondrial protein acetylome revealed the hyperacetylation of PDHA1 during sepsis

**Fig. 1:**
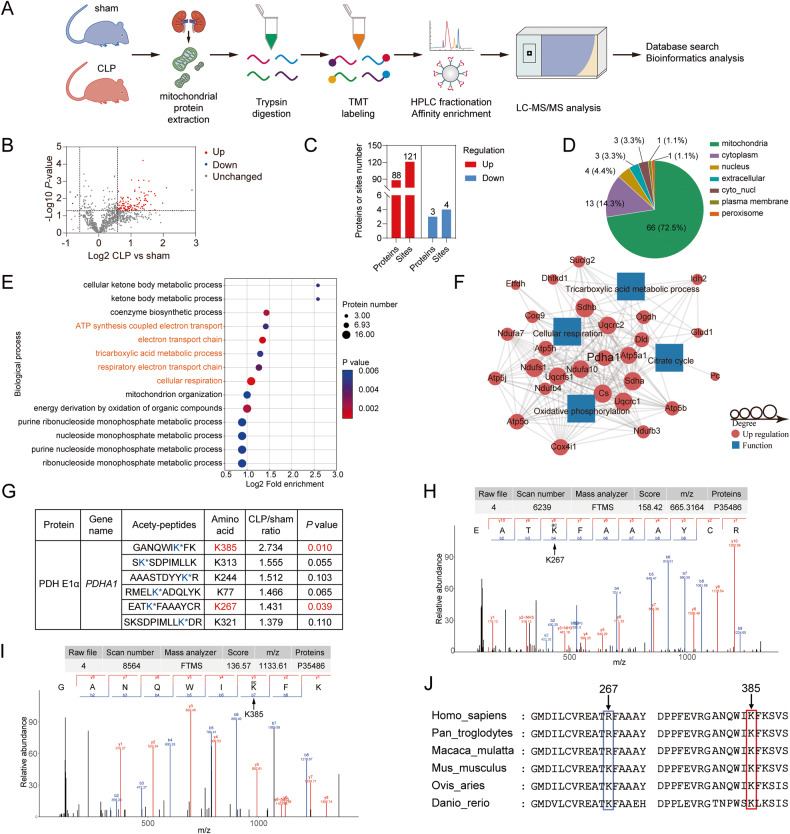
The renal mitochondrial protein acetylome identified PDHA1 hyperacetylation after CLP. **A** Flow chart showing the mitochondrial protein acetylome in the mouse kidney 8 h after CLP (*n* = 3). **B** Volcano plot showing the differentially modified proteins. **C** The number of differentially modified proteins. **D** Subcellular localization of the differentially modified proteins. **E** Biological processes of the differentially modified proteins. **F** Differentially modified proteins involved in cellular respiration, the tricarboxylic acid cycle, and oxidative phosphorylation. **G** Differential PDHA1 acetylated lysine sites. **H**, **I** Mass spectrometry spectrum of the acetylated PDHA1 K267 and K385 sites. **J** Protein multiple sequence alignment analysis of the PDHA1 267 and 385 sites.

Given that mitochondrial dysfunction is an important pathogenesis of SAKI and acetylation plays a pivotal role in regulating mitochondrial protein function [26–28], a Tandem Mass Tags (TMT)-labeled acetylome was used to identify changes in the acetylation of renal mitochondrial proteins 8 h after cecal ligation and puncture (CLP) in C57BL/6 mice (Fig. 1A). A total of 1135 acetylation sites on 525 proteins were identified, of which 1043 sites on 481 proteins contained quantitative information. The acetylation of 121 sites on 88 proteins was significantly upregulated in CLP group compared to sham group. The acetylation of 4 sites on 3 proteins was downregulated (Fig. 1B–D). Gene Ontology enrichment analysis showed that the main biological processes of the differentially modified proteins involved cellular respiration, tricarboxylic acid cycle (TCA), and oxidative phosphorylation (Fig. 1E). It was reported that fatty acid oxidation and TCA were inhibited, while glycolysis was enhanced in renal tubular epithelial cells during SAKI development [29–31]. Therefore, among the differential proteins involved in these metabolic processes, PDHA1, which is the first key rate-limiting enzyme between glycolysis and TCA, attracted our attention (Fig. 1F). There were 6 acetylation sites of PDHA1 containing quantitative information. However, only K385 and K267 were significantly different (Fig. 1G–I). Protein multiple sequence alignment analysis revealed that only the K385 site was relatively conserved among species (Fig. 1J). These results suggest that PDHA1 K385 acetylation may play an important role in SAKI.

### The downregulation of SIRT3 mediates PDHA1 hyperacetylation and inactivation, resulting in lactate overproduction

**Fig. 2:**
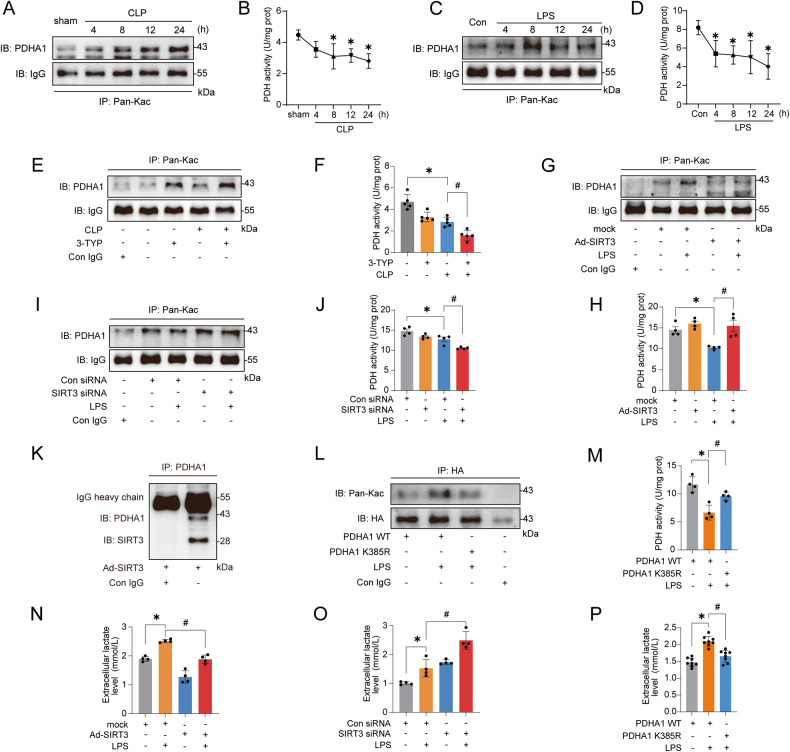
SIRT3 downregulation mediates PDHA1 hyperacetylation and inactivation, leading to lactate overproduction. **A**, **B** Changes in PDHA1 acetylation and PDH activity within 24 h after CLP in mice (*n* = 4). **C**, **D** Changes in PDHA1 acetylation and PDH activity in HK-2 cells within 24 h after LPS stimulation (*n* = 4). **E**, **F** Effects of SIRT3-specific inhibitor 3-TYP (5 mg/kg) treatment on CLP-induced PDHA1 hyperacetylation and PDH activity in mice (*n* = 5). **G**, **H** Effects of SIRT3 overexpression on LPS-induced PDHA1 hyperacetylation and PDH activity in HK-2 cells (*n* = 4). **I**, **J** Effects of SIRT3 knockdown on LPS-induced PDHA1 hyperacetylation and PDH activity in HK-2 cells (*n* = 4). **K** co-IP showing the interaction of SIRT3 with PDHA1 in HK-2 cells. **L**, **M** Effects of PDHA1 K385R mutation on PDHA1 acetylation and PDH activity in HK-2 cells (*n* = 4). **N**, **O** Effects of SIRT3 overexpression and SIRT3 knockdown on LPS-induced lactate production in HK-2 cells, respectively (*n* = 4). **P** Effects of PDHA1 K385R mutation on LPS-induced lactate production in HK-2 cells (*n* = 8). Data are mean ± SD; ^*^P, ^#^*P* < 0.05.

The results of immunoprecipitation (IP) confirmed the hyperacetylation of PDHA1 in the kidney after CLP (Fig. 2A). Since PDHA1 acetylation reduces PDH activity [6, 17], PDH activity was further assessed and the results demonstrated that in addition to the increase in PDHA1 hyperacetylation, PDH activity was decreased after CLP (Fig. 2B). Immunofluorescence analysis of the kidney revealed that PDHA1 was mainly and highly expressed in renal tubular epithelial cells (Fig. S1A). Therefore, lipopolysaccharide (LPS)-stimulated tubular cells, human kidney-2 (HK-2) cells, were used to establish the SAKI cell model. Consistently, PDHA1 was hyperacetylated and PDH activity was decreased after LPS stimulation (Fig. 2C, D). We next examined PDHA1 and SIRT3 protein expression in SAKI. The results showed that within 24 h of CLP induction in mice and LPS stimulation in HK-2 cells, PDHA1 expression did not change significantly, but SIRT3 expression was significantly downregulated (Fig. S1B–G). To clarify whether SIRT3 could regulate PDHA1 acetylation in SAKI, the mice were pretreated with the SIRT3-specific inhibitor 3-TYP before CLP, and 3-TYP further aggravated the PDHA1 hyperacetylation and PDH inactivation (Fig. 2E, F). In vitro, we overexpressed or knocked down the expression of SIRT3 in HK-2 cells, respectively (Fig. S1H–L). SIRT3 overexpression inhibited the LPS-induced PDHA1 hyperacetylation and PDH inactivation, while SIRT3 knockdown reversed this effect (Fig. 2G–J). These data suggest that SIRT3 downregulation contributes to PDHA1 hyperacetylation and PDH inactivation. Molecular docking between SIRT3 and PDHA1 revealed that multiple groups of residues were involved in hydrogen bond formation, including PDHA1 K385 and SIRT3 R158 (Fig. S1M and Table S1). Additionally, PDHA1 K385 was shown to engage in electrostatic interactions with SIRT3 D156 (Fig. S1M, Table S1). Importantly, the interaction between SIRT3 and PDHA1 was further confirmed by co-IP in HK-2 cells (Fig. 2K). Finally, we constructed HA-tagged PDHA1 wild-type (PDHA1 WT) and mutant plasmids (PDHA1 K385R). The PDHA1 K385R mutant was designed to mutate the 385th lysine into arginine to mimic the deacetylation of PDHA1 K385 (Fig. S1N, O). LPS stimulation enhanced PDHA1 WT hyperacetylation and PDH inactivation, whereas the acetylation level of PDHA1 K385R was decreased and PDH activity was recovered (Fig. 2L, M).

We next examined whether PDHA1 inactivation promotes lactate overproduction during SAKI. It was showed that 8-24 h after CLP in mice, not only serum creatinine (SCr) and urea nitrogen (BUN) were significantly increased, but also renal lactate levels were significantly elevated (Fig. S1P–R). Pretreatment with 3-TYP significantly increased renal lactate levels after CLP (Fig. S1S). In vitro, lactate production in HK-2 cells was also significantly increased after LPS stimulation (Fig. S1T). However, activating PDHA1 with sodium dichloroacetate (DCA, a PDHA1 activator) pretreatment or SIRT3 overexpression reversed the LPS-induced increase in lactate (Figs. S1U and 2N). Conversely, inactivating PDHA1 by 3-TYP pretreatment or SIRT3 knockdown further promoted the increase in lactate (Figs. S1V, W and 2O). Furthermore, HK-2 cells transfected with PDHA1 K385R attenuated LPS-induced lactate increase in PDHA1 WT (Fig. 2P). In summary, PDHA1 hyperacetylation and inactivation promote lactate production in SAKI.

### Increased Lactate is Associated with a High Incidence of SAKI and Poor Prognosis in Clinical Sepsis Patients

**Fig. 3:**
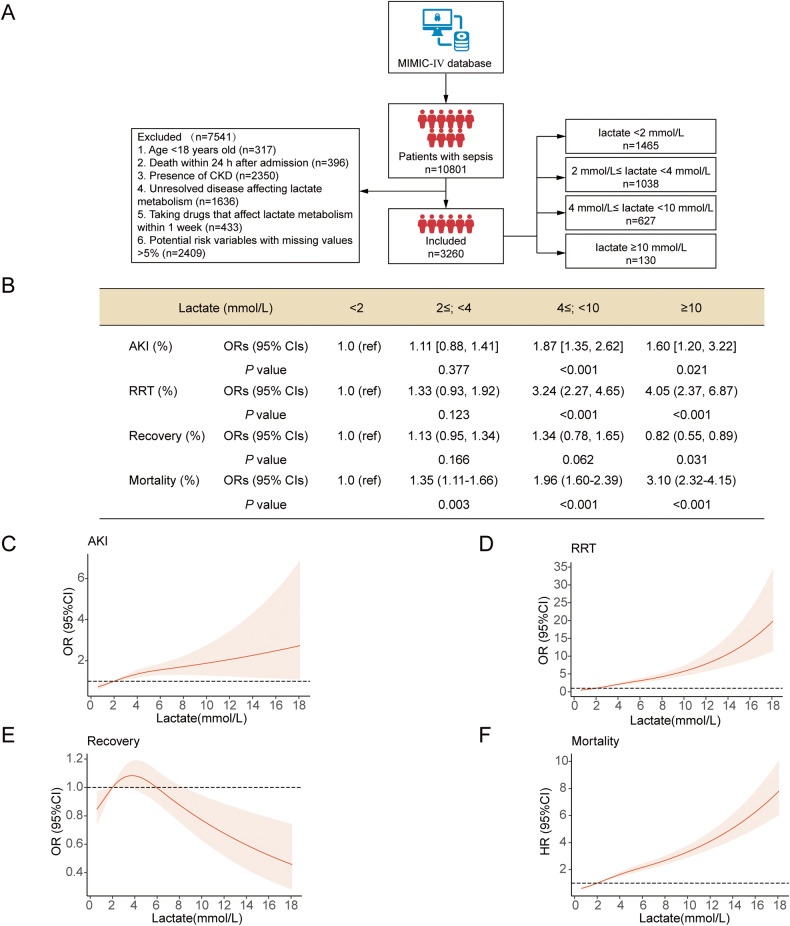
High blood lactate levels are associated with a high incidence of AKI and poor prognosis in septic patients. **A** Flow chart showing the retrospective analysis of sepsis patients with different lactate levels in the MIMIC-IV database. **B** The ORs (95% CIs) for the occurrence, RRT requirements, and recovery of AKI and HRs (95% CIs) for mortality across the groups. Regression models were adjusted for confounding variables selected from univariate analysis, including age, gender, ethnicity, admission type, liver disease, white blood cell, baseline creatinine, volume, and SOFA score. **C**–**F** Concentration-response relationship between lactate and outcomes. Restricted cubic spline curves were used to evaluate the association between concentration of lactate and the occurrence, RRT requirements, recovery, as well as in-hospital mortality of AKI in septic patients.

Increased lactate is an independent risk factor for SAKI [32, 33]. However, no research has explored the relationship between different lactate levels and SAKI. A retrospective cohort study of the Multiparameter Intelligent Monitoring in Intensive Care Database IV (MIMIC-IV, a published and authorized online database [34]) was performed to clarify the clinical importance of different lactate levels in SAKI. A total of 3260 patients with sepsis were included. According to their blood lactate levels (mmol/L) "<2", "2≤; < 4", "4≤; < 10", "≥10", the patients were divided into 4 groups (Fig. 3A). With adjusting confounding variables selected from univariate analysis, we found that when lactate was ≥4 mmol/L, the incidence of SAKI and RRT usage rate increased significantly compared with when lactate was <2 mmol/L (Fig. 3B–D). When lactate was ≥ 10 mmol/L, lactate levels were significantly associated with a decrease in the recovery rate of SAKI (Fig. 3B, E). When blood lactate was ≥2 mmol/L, lactate levels were significantly associated with increased in-hospital mortality (Fig. 3B, F). More detailed patient characteristics, other shock-related parameters, other SAKI-related parameters, confounding variables selected from univariate analysis, and clinical outcomes are shown in Supplementary Materials (Tables S2–S5 and Fig. S2A, B).

### The increase of lactate aggravates SAKI

**Fig. 4:**
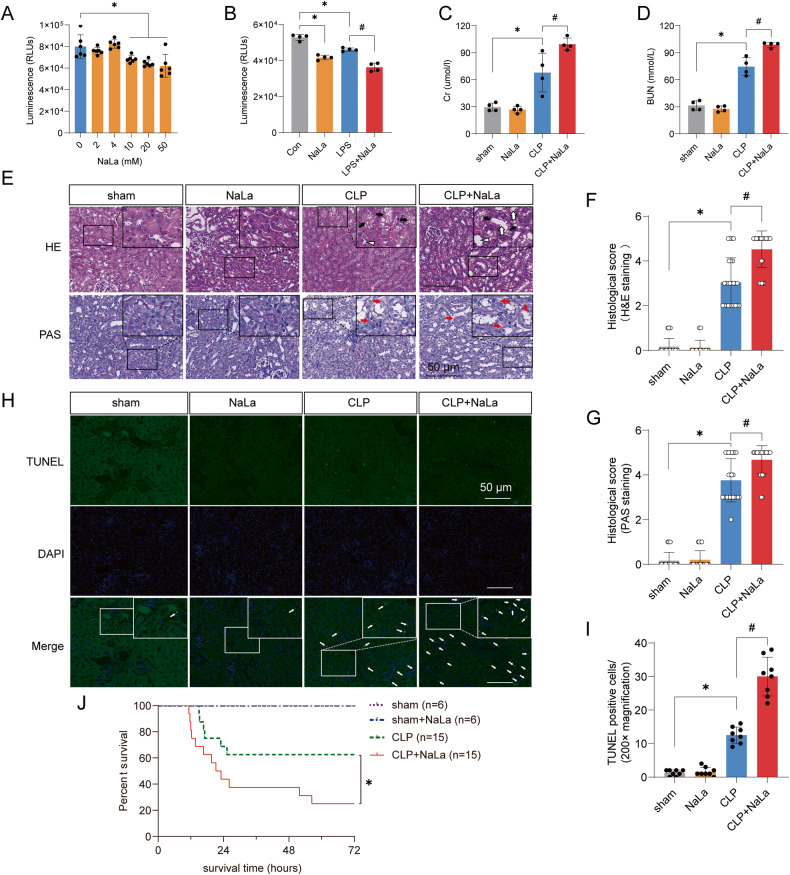
High lactate levels directly contribute to SAKI aggravation. **A** Effects of different concentrations (0 ~ 50 mM) of lactate on the viability of HK-2 cells (*n* = 6). **B** Lactate (10 mM) treatment significantly promoted the LPS-induced decrease in cell viability (*n* = 4). **C**, **D** Lactate (1 g/kg) pretreatment significantly increased CLP-induced increases in SCr and BUN in mice (*n* = 4). **E**–**G** Haematoxylin-eosin (HE) staining (upper panel) and periodic acid-Schiff (PAS) staining (lower panel) of the kidney following CLP-induced sepsis (*n* = 25). Black arrow: renal tubular vacuoles, structural disorder; white arrow: brush border loss; white triangle: tubular lumen dilation; red arrow: renal tubular basement membrane rupture. Scale bars, 50 μm. **H**, **I** Cell apoptosis in the kidney was examined by TUNEL staining (*n* = 8). Scale bars, 50 μm. **J** The survival rates in the sham, sham+lactate, CLP, and CLP+lactate groups. Data are mean ± SD; ^*^P, ^#^*P* < 0.05.

To explore whether the increase of lactate directly exacerbates SAKI, the HK-2 cells and mice were pretreated with exogenous lactate (sodium lactate, NaLa). The results showed that lactate ≥ 10 mM significantly reduced HK-2 cell viability (Fig. 4A), which may explain why when septic patients lactate was ≥ 10 mM, not only the incidence of SAKI and RRT usage rate increased, but also the recovery rate of SAKI decreased. We further revealed that lactate could exacerbate the LPS-induced decrease in cell viability (Fig. 4B). In vivo experiments showed that lactate alone did not cause organ damage, which may be due to the normal activity of PDH under physiological conditions so that high levels of lactate could be metabolized by the body. However, the addition of exogenous lactate further aggravated the increases in SCr and BUN induced by CLP in mice and aggravated histopathological damage and apoptosis in the kidney (Fig. 4C–I). Furthermore, lactate reduced the survival rates of CLP mice (Fig. 4J). In conclusion, the increase of lactate can directly promote SAKI.

### The lactylome revealed an increase in the lactylation of Fis1 after sepsis

**Fig. 5:**
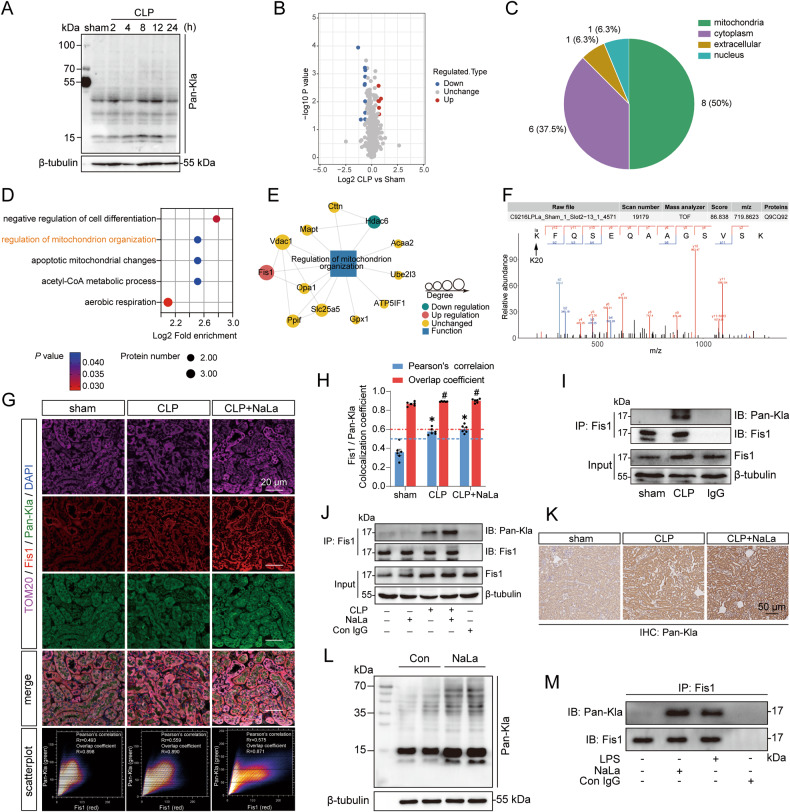
The lactylome revealed that renal Fis1 lactylation was elevated after sepsis. **A** Changes in Pan-Kla in mouse kidneys within 24 h after CLP. **B** Volcano plot showing the differentially lactylated proteins in the kidney 8 h after CLP (*n* = 3). **C** The number and intracellular localization of differentially lactylated proteins. **D** Biological processes of the differentially lactylated proteins. **E** Differentially lactylated proteins involved in the regulation of mitochondrion organization. **F** Mass spectrometry spectrum of the lactylated Fis1 K20 sites. **G**, **H** Fis1, Pan-Kla, and mitochondrial marker TOM20 immunofluorescent triple staining showing the co-localization of Fis1 and Pan-Kla. The images were analyzed by the Colocalization Finder plugin of ImageJ software (*n* = 6). Fis1 and PanKla were considered co-localized when the overlap coefficient was >0.6 and Pearson’s correlation coefficient was >0.5. Scale bars, 20 μm. **I**, **J** Changes in renal Fis1 lactylation following CLP or lactate (1 g/kg) administration in mice. **K** Immunohistochemistry showing the changes in Pan-Kla in mouse renal tissue after CLP and lactate administration. **L** Changes in Pan-Kla in HK-2 cells after lactate stimulation. **M** Changes in Fis1 lactylation after LPS or lactate stimulation in HK-2 cells. Data are mean ± SD; ^*^P, ^#^*P* < 0.05.

Given that lactate plays an important role in SAKI and that lactylation is associated with the regulation of protein function [19], we assessed whether lactylation is involved in the development of SAKI. Renal lactylation was examined with the pan lactyl-lysine (Pan-Kla) antibody, and the results showed that the lactylation level in the mouse kidney was obviously changed after CLP (Fig. 5A). Thus, a 4D-label free lysine lactylome was used to analyze the changes in lactylation in kidneys 8 h after CLP. The results revealed that fifty percent of differentially modified proteins were located in mitochondria (Fig. 5B, C). Further functional enrichment analysis revealed that differentially modified proteins were involved in the regulation of mitochondrial organization (Fig. 5D). We focused on mitochondrial fission 1 protein (Fis1), which is a mitochondrial network regulator, and was the top1 protein with increased lactylation (Fig. 5E, F). The co-localization of Fis1 with Pan-Kla was observed in the kidneys of CLP mice with and without lactate treatment (Fig. 5G, H). The IP results showed that Fis1 lactylation was significantly increased after CLP, and supplementation with exogenous lactate boosted the CLP-induced increase in Fis1 lactylation (Fig. 5I, J).

The immunohistochemistry results showed that the changes in lactylation in renal tubular epithelial cells were obvious (Fig. 5K). The western blot results indicated that lactate could definitely increase Pan-Kla levels of HK-2 cells (Fig. 5L). Thereafter, it was confirmed that Fis1 was located in the mitochondria of HK-2 cells (Fig. S2C). Importantly, both lactate and LPS stimulation could significantly increase Fis1 lactylation in HK-2 cells (Fig. 5M). These results reveal that Fis1 lactylation may play an important role in SAKI.

### Lactate mediates excessive mitochondrial fission to exacerbate SAKI

**Fig. 6:**
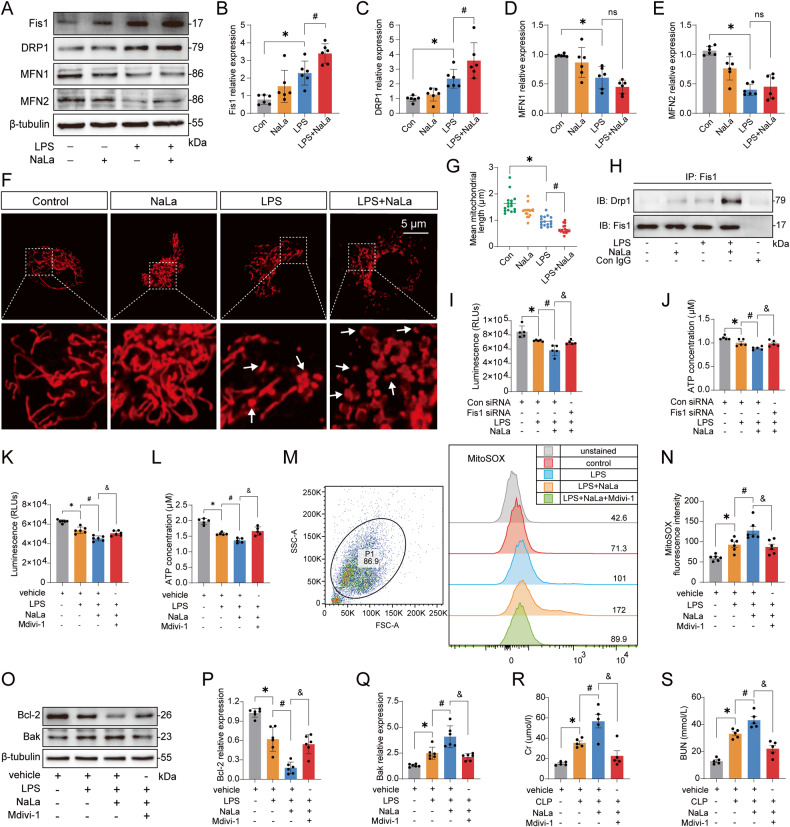
Lactate aggravates SAKI by promoting excessive mitochondrial fission. **A**–**E** Effects of lactate on the protein expression of Fis1, DRP1, MFN1, and MFN2 in HK-2 cells (*n* = 6). **F**–**G** The mitochondrial morphology of HK-2 cells was observed under confocal microscope by MitoTracker staining. The images were analyzed by the Mitochondrial Network Analysis (MiNA) plugin of ImageJ software (*n* = 17). Scale bars, 5 μm. **H** Lactate promoted the LPS-induced Fis1-DRP1 interaction in HK-2 cells. **(I-J)** Fis1 knockdown attenuated the reduction in cell viability and ATP depletion induced by lactate+LPS stimulation in HK-2 cells (*n* = 5). **K**–**L** Mdivi-1 (10 μM) administration attenuated the reduction in cell viability and ATP depletion induced by lactate+LPS stimulation in HK-2 cells (*n* = 5). **M**, **N** Flow cytometry assessment after MitoSOX staining showed that Mdivi-1 reduced the LPS+lactate-induced mtROS elevation in HK-2 cells (*n* = 6). **O**–**Q** The effects of Mdivi-1 on the expression of the antiapoptotic protein Bcl-2 and the proapoptotic protein Bak in HK-2 cells (*n* = 6). **R**, **S** Mdivi-1 reduced CLP+lactate-induced elevation of SCr and BUN in mice (*n* = 5). Data are mean ± SD; ^*^P, ^#^*P*, ^&^*P* < 0.05.

Fis1 is localized to the mitochondrial outer membrane, and participates in mitochondrial network regulation [21, 35–37]. This finding inspired us to assess the role of Fis1 lactylation in regulating the mitochondrial network in the pathogenesis of SAKI. We measured the expression of the mitochondrial fission proteins Fis1 and DRP1, as well as the fusion proteins mitofusin 1 (MFN1) and mitofusin 2 (MFN2) in vivo and in vitro. Fis1 and DRP1 were significantly upregulated, while MFN1 and MFN2 were significantly downregulated after SAKI (Fig. S3A–J). It implied that the mitochondrial fragmentation was increased. Lactate alone only slightly affected the expression of Fis1, DRP1, MFN1, and MFN2, with no statistical difference. However, when lactate was combined with LPS to stimulate HK-2 cells, lactate further enhanced the expression of Fis1 and DRP1 but did not affect MFN1 and MFN2 expression (Fig. 6A–E). These findings indicated that the increase of lactate promoted excessive mitochondrial fission. To verify this result, we observed mitochondrial morphological changes using MitoTracker staining. Lactate significantly promoted LPS-induced shortening of the mean mitochondrial length (Fig. 6F, G). Previous studies have shown that Fis1, which is an adaptor protein, can interact with the fission executor protein DRP1 to promote mitochondrial fission [24, 25]. We showed that Fis1 interacted with DRP1 after LPS stimulation, and additional lactate treatment further enhanced this effect (Fig. 6H).

Next, we determined the role of excessive mitochondrial fission in SAKI. First, lactate worsened the LPS-induced decrease in ATP levels (Fig. S3K). However, Fis1 knockdown attenuated the reduction in cell viability and ATP depletion induced by lactate plus LPS stimulation (Figs. S3L, M and 6I, J). Second, compared with vehicle + LPS + lactate group, the mitochondrial fission inhibitor Mdivi-1 improved cell viability and increased ATP levels (Fig. 6K, L). Moreover, Mdivi-1 inhibited mtROS overproduction induced by lactate plus LPS stimulation (Fig. 6M, N). Lactate plus LPS stimulation inhibited the expression of the antiapoptotic protein Bcl-2 and promoted the expression of the proapoptotic protein Bak. However, Mdivi-1 administration abrogated this effect (Fig. 6O–Q). Finally, Mdivi-1 significantly reduced the SCr and BUN levels, and inhibited renal cell apoptosis compared with vehicle+CLP+lactate group (Figs. 6R, S, S3N, O). Moreover, it also inhibited 4-Hydroxynonenal (4-HNE, an index of oxidative stress-induced lipid peroxidation) expression (Fig. S3P, Q). These findings suggest that high levels of lactate-mediated excessive mitochondrial fission promote ATP depletion, mtROS overproduction, and mitochondrial apoptosis, thereby exacerbating SAKI.

### Fis1 lysine 20 lactylation plays a pivotal role in SAKI

**Fig. 7:**
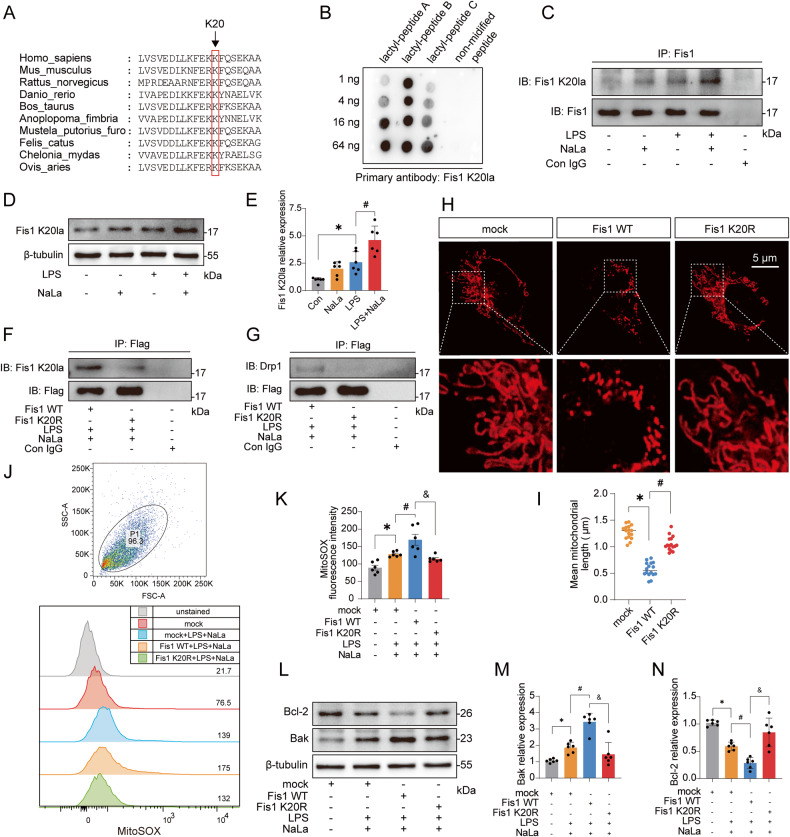
Fis1 K20 is the key site by which lactate mediates Fis1 lactylation and exacerbates SAKI. **A** Protein multiple sequence alignment analysis of Fis1 20 sites. **B** Verification of the specificity of the Fis1 K20la antibody by dot blot experiment. **C** Immunoblotting with the Fis1 K20la antibody after Fis1 IP in HK-2 cells. **D**, **E** Recognition of Fis1 lactylation in HK-2 whole-cell lysates by the Fis1 K20la antibody (*n* = 6). **F** Fis1 K20R mutation eliminated Fis1 K20la in HK-2 cells. **G** Fis1 K20R mutation inhibited the Fis1 interaction with DRP1 in HK-2 cells. **H**, **I** Effects of Fis1 WT and K20R plasmid overexpression on the mitochondrial network (*n* = 16). Scale bars, 5 μm. **J**, **K** Fis1 K20R mutation inhibited Fis1 WT-mediated mtROS overproduction (*n* = 6). **L**–**N** Fis1 K20R mutation inhibited the Fis1 WT-mediated Bcl-2 expression decrease and Bak expression increase (*n* = 6). Data are mean ± SD; ^*^P, ^#^*P*, ^&^*P* < 0.05.

The lactylome showed that lactylation at lysine 20 of Fis1 (Fis1 K20la) was significantly elevated after CLP (Fig. 5F). Further protein multiple sequence alignment analysis showed that lysine 20 of Fis1 was relatively conserved among species (Fig. 7A). Thus, we customized a Fis1 K20la-specific antibody and verified that the Fis1 K20la antibody could specifically recognize the Fis1 lactyl-peptide (Fig. 7B). Detailed peptide information can be found in Fig. S4A–E. It was further confirmed that the Fis1 K20la antibody could recognize Fis1 K20la after IP of Fis1 or using whole-cell lysates (Fig. 7C–E).

To understand the role of Fis1 K20la in SAKI, we constructed wild-type (Fis1 WT) and mutant plasmids of Fis1. The Fis1 mutant plasmid was used to mutate the 20th lysine into arginine (Fis1 K20R) to mimic the delactylation of Fis1 K20 (Fig. S4F–I). Compared with that of Fis1 WT, the lactylation of Fis1 K20R was eliminated (Fig. 7F). Importantly, Fis1 K20R significantly reduced the interaction of Fis1 with DRP1 (Fig. 7G). Morphologically, compared with that in the mock group, Fis1 WT significantly promoted mitochondrial fission, while Fis1 K20R rescued this effect (Fig. 7H, I). Finally, overexpression of the Fis1 WT plasmid significantly reduced cell viability and ATP levels, promoted mtROS overproduction, and decreased Bcl-2 while upregulating Bak expression (Figs. S4J, K, 7J–N). However, none of these effects were observed in response to the Fis1 K20R overexpression plasmid. These data indicate that reducing Fis1 K20la plays an important role in alleviating SAKI.

### Decreasing lactate and Fis1 lactylation attenuates SAKI

**Fig. 8:**
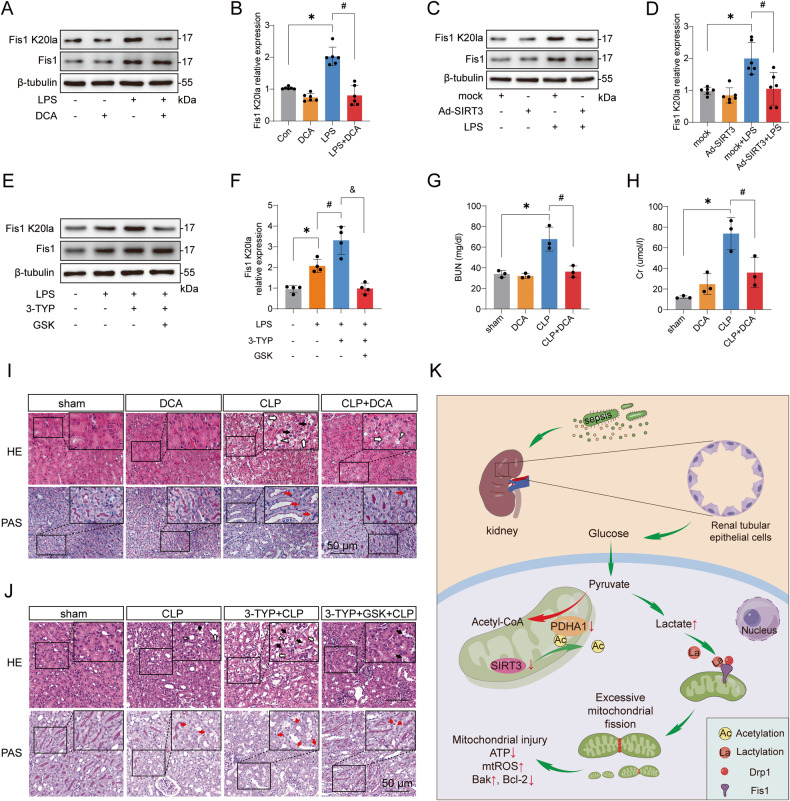
The effects of reducing lactate and Fis1 lactylation on SAKI. **A**, **B** Effects of DCA (5 mM) treatment on Fis1 K20la level in LPS-stimulated HK-2 cells (*n* = 6). **C**, **D** Effects of SIRT3 overexpression on Fis1 K20la level in LPS-stimulated HK-2 cells (*n* = 6). **E**, **F** Effects of 3-TYP (50 μM) and GSK (5 μM) treatment on Fis1 K20la level in LPS-stimulated HK-2 cells (*n* = 4). **G**, **H** Effects of intraperitoneal injection of DCA (25 mg/kg) on SCr and BUN in CLP mice (*n* = 3). **I** Effects of intraperitoneal injection of DCA on renal pathological damage in CLP mice. Black arrow: nuclei of renal tubular cells shed to the lumen; White arrow: renal tubular vacuoles, structural disorder; White triangle: brush border loss; Red arrow: renal tubular basement membrane rupture. Scale bars, 50 μm. **J** Effects of intraperitoneal injection of 3-TYP (5 mg/kg) and GSK (20 mg/kg) on renal pathological damage in CLP mice. Scale bars, 50 μm. **K** Schematic of PDHA1 hyperacetylation-mediated lactate overproduction promoting SAKI via Fis1 lactylation. Data are mean ± SD; ^*^P, ^#^*P* < 0.05.

Since PDHA1 activation by DCA or SIRT3 overexpression could reduce lactate levels in HK-2 cells (Figs. S1U, 2N), we then determined whether this effect could reduce Fis1 K20la levels. The results revealed that both DCA and SIRT3 overexpression significantly reduced Fis1 K20la levels (Fig. 8A–D). In contrast, inhibition of SIRT3 with 3-TYP or knockdown of SIRT3 expression promoted lactate production and enhanced Fis1 K20la levels (Figs. S1W, 2O and S5A–D). However, treatment with GSK2837808A (GSK), a lactate dehydrogenase inhibitor to reduce lactate production, attenuated 3-TYP-mediated enhancement of Fis1 K20la (Fig. 8E, F).

Furthermore, DCA treatment and SIRT3 overexpression and subsequent decrease of Fis1 K20la relieved the LPS-induced reductions in cell viability and ATP levels (Fig. S5E–H). GSK treatment rescued 3-TYP-mediated decrease of cell viability and ATP levels in LPS-challenged HK-2 cells (Fig. S5I, J). In vivo, the administration of DCA significantly reduced CLP-induced increases in SCr and BUN and improved renal pathological damage (Figs. 8G–I and S5K, L). Finally, GSK treatment significantly alleviated 3-TYP-mediated SAKI aggravation (Figs. 8J and S5M–P). This evidence suggests that reducing lactate and Fis1 lactylation can alleviate SAKI.

## Discussion

Protein acetylation plays an important role in regulating mitochondrial metabolism. SIRT3 is the most important deacetylase that regulates mitochondrial protein acetylation. Studies have shown that SIRT3 deacetylates PDHA1 and enhances PDH activity, thereby mediating cellular metabolic reprogramming in tumor cells [6, 38]. In this study, we demonstrated that SIRT3 downregulation led to PDHA1 hyperacetylation in SAKI. PDHA1 has multiple acetylation sites, and the specific acetylation site may be different under different pathophysiological conditions. High salt intake increases PDHA1 K83 acetylation in adipocytes to regulate adaptive thermogenesis [39]. In tumor studies, SIRT3 deacetylated PDHA1 K321 to enhance PDH activity and increase oxidative phosphorylation flux [6, 38]. In addition, PDHA1 K336 hyperacetylation could reduce PDH activity, resulting in impaired glucose oxidation in skeletal muscle [8]. Our acetylome data revealed that acetylation at PDHA1 K267 and K385 was significantly elevated in SAKI, which is similar to the results reported by Yu Zhang et al. during renal fibrosis [17]. However, only PDHA1 K385 was highly conserved among species and the acetylation of PDHA1 K385 was involved in PDH activity regulation in SAKI development.

In sepsis, PDH inactivation was observed in a variety of cells, including peripheral blood mononuclear cells, skeletal muscle cells, and vascular endothelial cells [10, 13, 40]. PDHA1 hyperacetylation is an important mechanism of PDH inactivation [6, 8, 17]. Our study showed that PDHA1 hyperacetylation and inactivation in renal tubular cells were accompanied by lactate overproduced in the development of SAKI. Altering PDHA1 acetylation levels could modulate lactate production. As emerging studies found that renal oxygen delivery/consumption were not significantly changed during SAKI, hypoxia could not fully explain lactate overproduction in kidney [41, 42]. Metabolic reprogramming has been described in SAKI [43]. It was reported that fatty acid oxidation and TCA were inhibited, while glycolysis was enhanced and accompanied lactate overproduction in renal tubular epithelial cells during SAKI [29–31]. The present study implies that the PDHA1 hyperacetylation and inactivation may be an important reason for the glycolysis enhancement and TCA inhibition.

In addition to being a metabolite of glycolysis, lactate can selectively stimulate the release of Mg^2+^ to promote mitochondrial dysfunction [44]. Furthermore, studies have evidenced that lactate overproduction in sepsis can promote inflammatory responses, induce vascular hyperpermeability, and inhibit autophagy [45–48]. In recent years, lactate was shown to be involved in the lactylation of proteins [19]. Since the discovery of lactylation, scholars have explored the functional mechanisms of histone and nonhistone lactylation in different diseases such as sepsis and tumors [49, 50]. However, most of the current studies have reported histone lactylation, but very little is known about nonhistone lactylation. To our knowledge, only macrophage-derived nonhistone HMGB1 lactylation has been reported in sepsis [49]. We demonstrated that lactylation of the nonhistone Fis1 was significantly increased after CLP. The 20th lysine plays a pivotal role in Fis1 lactylation. In fact, the sources of renal lactate after sepsis in vivo are renal cells and circulating blood. In vitro, we demonstrated that PDHA1 hyperacetylation and inactivation mediated the increase in lactate in LPS-stimulated renal tubular epithelial cells. Both this increase in endogenous lactate and supplementation with exogenous lactate can promote Fis1 K20la levels in renal tubular epithelial cells. This may partly explain why clinically high blood lactate levels are associated with a high incidence of SAKI and poor prognosis in sepsis patients. The increase in Fis1 K20la could further promote excessive mitochondrial fission. This effect was mainly due to the interaction of Fis1 with DRP1. Originally, Fis1 was thought to act as a DRP1 adaptor in yeast [51]. However, some studies in mammalian cells later showed no direct interaction between Fis1 and DRP1 [52]. There is still much evidence to support the interaction of Fis1 and DRP1 [23–25]. As recently reported by Shiyuan Wang et al., phosphorylated Fis1 interacts with DRP1 to promote mitochondrial fission [22]. Unlike mitochondrial fission factor (MFF), which is another adaptor of DRP1, Fis1 mediates mitochondrial peripheral fission under stress conditions, whereas MFF mediates midzone fission for mitochondrial biogenesis during cell proliferation [21]. Additionally, it was found that Fis1 regulated fission by interacting with MFN1, MFN2, OPA1, and TBC1D15 [36, 37, 53]. However, the interaction of Fis1 with these proteins was not detected in our SAKI model.

Moderate mitochondrial fission is part of mitochondrial quality control, and these mechanisms favor the removal of damaged mitochondria, while excessive mitochondrial fission promotes cellular injury [20, 21]. This may be because when mitophagy is insufficient to clear mitochondrial fragmentation, excessive mitochondrial fission will lead to ATP depletion, massive production of mtROS, and mitochondrial apoptosis [20]. Our previous study reported that autophagy activation is insufficient in SAKI [54]. To date, the pathogenesis of SAKI mainly includes inflammation, microcirculation dysfunction, abnormal cell response, including cell cycle arrest and metabolic reprogramming [27, 55]. Inflammation also mediates excessive oxidative stress and renal tubular cell apoptosis [56]. The present study reveals that elevated Fis1 K20la promotes excessive mitochondrial fission, which results in ATP depletion, mtROS overproduction, and mitochondrial apoptosis. Inhibiting mitochondrial fission with Mdivi-1 reversed these effects. It suggests the important roles of mitochondrial dysfunction, energy depletion, oxidative stress, and apoptosis of renal tubular cells in SAKI development.

It is extremely difficult to obtain renal specimens from patients with sepsis since SAKI is not an indication for renal biopsy in clinical practice, and kidney biopsy during sepsis carries the risk of infection spreading as well. Therefore, an important limitation is the lack of confirmation regarding the changes in renal lactate and Fis1 K20la in septic patients, as we only observed their increase after CLP in mice and in LPS-stimulated HK-2 cells.

In conclusion, our data shed light on the relationship between nonhistone Fis1 lactylation and SAKI. Most importantly, our findings reveal a new mechanism linking lactate and organ damage in sepsis and emphasize the importance of therapeutic strategies to reduce blood lactate levels in patients with sepsis.

## Materials and methods

### Animal study

Male C57BL/6 mice (weighing 19–23 g, 6–8 weeks old) were obtained from the Animal Experimental Center of Southern Medical University. The experiments were approved by the Animal Care Committee of the Southern Medical University of China and were performed according to the National Institutes of Health guidelines for ethical animal treatment. The CLP procedure was performed as described in a previous protocol [57]. 5 mg/kg 3-TYP (S8628, Selleck), 25 mg/kg DCA (S8615, Selleck), 1 g/kg NaLa (71718, Sigma), and 20 mg/kg GSK (HY-100681, MCE) were intraperitoneally injected 0.5 h before CLP as needed. Mdivi-1 (50 mg/kg, S7162, Selleck) was intraperitoneally injected 1 h before CLP.

## Supplementary information

Supplementary figure legends

Fig. S1

Fig. S2

Fig. S3

Fig. S4

Fig. S5

Supplementary Materials

Original Data File

## Data Availability

Source data are provided with this paper. All other data associated with this study are presented in the paper, the Supplementary Figures or the Supplementary Materials.
