# Supplementary material for: PDHA1 hyperacetylation-mediated lactate overproduction promotes sepsis-induced acute kidney injury via Fis1 lactylation
Source: Cell Death Dis. 2023 Jul 21;14(7):457. doi: 10.1038/s41419-023-05952-4 (PMC10362039; doi:10.1038/s41419-023-05952-4)
Supplement: Supplementary file 1 — Supplementary figure legends [file 41419_2023_5952_MOESM1_ESM.docx]

**Supplementary figure legends：**

**Fig. S1 Effects of SIRT3 expression level on PDHA1 acetylation and activity, and lactate production. (A)** Immunofluorescence showing the expression of PDHA1 in mouse kidney. White circle: glomerulus. **(B-D)** Changes in renal SIRT3 and PDHA1 expression within 24 h after CLP (n=5). **(E-G)** Changes in SIRT3 and PDHA1 expression in HK-2 cells within 24 h after LPS challenge (n=4-5). **(H)** Ad-SIRT3 overexpression validation in HK-2 cells. Green fluorescence represents GFP. Scale bars, 50 μm. **(I-J)** The expression of SIRT3 and Flag-SIRT3 after Ad-SIRT3 transfection in HK-2 cells (n=4). **(K-L)** The expression of SIRT3 after SIRT3 siRNA transfection (n=3). **(M)** A docking model showing the interactions between SIRT3 and PDHA1. PDHA1 was a deep blue cartoon model, while SIRT3 was s a cyan cartoon model. Their binding sites were visualized as stick structures. **(N-O)** Verification of PDHA1 WT and K385R plasmid overexpression in HK-2 cells. **(P-Q)** Changes of SCr and BUN within 24 hours after CLP in mice (n=4). **(R)** Changes of renal lactate levels within 24 h after CLP in mice (n=4). **(S)** Effects of intraperitoneal injection of 3-TYP (5 mg/kg) on renal lactate levels in CLP mice (n=5). **(T)** Extracellular lactate levels in HK-2 cells within 24 h after LPS stimulation (n=3). **(U)** Effects of DCA (5 mM) treatment on lactate levels in LPS-stimulated HK-2 cells (n=4). **(V-W)** Effects of 3-TYP (50 μM) treatment on PDH activity and lactate levels in LPS-stimulated HK-2 cells (n=4). Data are mean±SD; ^*^*P*, ^#^*P* < 0.05; ns, not significant.

**Fig. S2** **The SCr and BUN levels of SAKI and Non-SAKI patients, and mitochondrial localization of Fis1.** **(A-B)** Changes in the levels of SCr and BUN between SAKI and Non-SAKI patients at ICU admission, maximum during ICU, and ICU discharge. Data are mean±SD; ^*^*P* < 0.05, vs. Non-SAKI. **(C)** Western blotting showing the mitochondrial localization of Fis1 in HK-2 cells.

**Fig. S3 Lactate aggravates SAKI by promoting excessive mitochondrial fission.** **(A-E)** Fis1, DRP1, MFN1, and MFN2 protein expression within 24h after CLP in mice (n=4-6). **(F-J)** Fis1, DRP1, MFN1, and MFN2 protein expression within 24h after LPS stimulation in HK-2 cells (n=4-6). **(K)** Lactate promoted the LPS-induced decrease in ATP levels in HK-2 cells (n=6). **(L-M)** Expression of Fis1 in HK-2 cells transfected with Fis1 siRNA (n=3). **(N-O)** Cell apoptosis in the kidney was examined by TUNEL staining (n=8). Scale bars, 50 μm. **(P-Q)** Representative images and quantification of 4-HNE staining in kidney specimens (n=10). Scale bars, 20 μm. Data are mean±SD; ^*^*P*, ^#^*P*, ^&^*P* < 0.05.

**Fig. S4 The role of Fis1 K20la in SAKI. (A)** Three lactylated antigenic peptides and 1 non-modified antigenic peptide for antibody synthesis. **(B-E)** Mass spectrometry spectrum of the 3 lactylated peptides and 1 non-modified peptide. **(F-I)** Verification of Fis1 WT and K20R plasmid overexpression (n=3). **(J-K)** The Fis1 K20R mutation inhibited Fis1 WT-mediated decrease in HK-2 cell viability and ATP levels (n=6). Data are mean±SD; ^*^*P*, ^#^*P*, ^&^*P* < 0.05.

**Fig. S5 The effects of changing lactate production and Fis1 K20la level on SAKI. (A-B)** Effects of 3-TYP (50 μM) treatment on Fis1 K20la level in LPS-stimulated HK-2 cells (n=6). **(C-D)** Effects of SIRT3 knockdown on Fis1 K20la level in LPS-stimulated HK-2 cells (n=6). **(E-H)** Effects of DCA (5 mM) treatment and SIRT3 overexpression on cell viability and ATP content in LPS-stimulated HK-2 cells (n=6).

**(I-J)** Effects of 3-TYP (50 μM) and GSK (5 μM) treatment on cell viability and ATP content in LPS-stimulated HK-2 cells (n=8). **(K-L)** H&E staining and PAS staining pathological score of kidneys in CLP mice treated with DCA (25 mg/k) (n=25). **(M-N)** Effects of the treatment with 3-TYP (5 mg/kg) and GSK (20 mg/kg) on SCr and BUN in CLP mice (n=5). **(O-P)** H&E staining and PAS staining pathological score of kidneys in CLP mice treated with 3-TYP (5 mg/kg) and GSK (20 mg/kg) (n=25). Data are mean±SD; ^*^*P*, ^#^*P* < 0.05.
