# Supplementary figures and images for: PDHA1 hyperacetylation-mediated lactate overproduction promotes sepsis-induced acute kidney injury via Fis1 lactylation
Source: Cell Death Dis. 2023 Jul 21;14(7):457. doi: 10.1038/s41419-023-05952-4 (PMC10362039; doi:10.1038/s41419-023-05952-4)

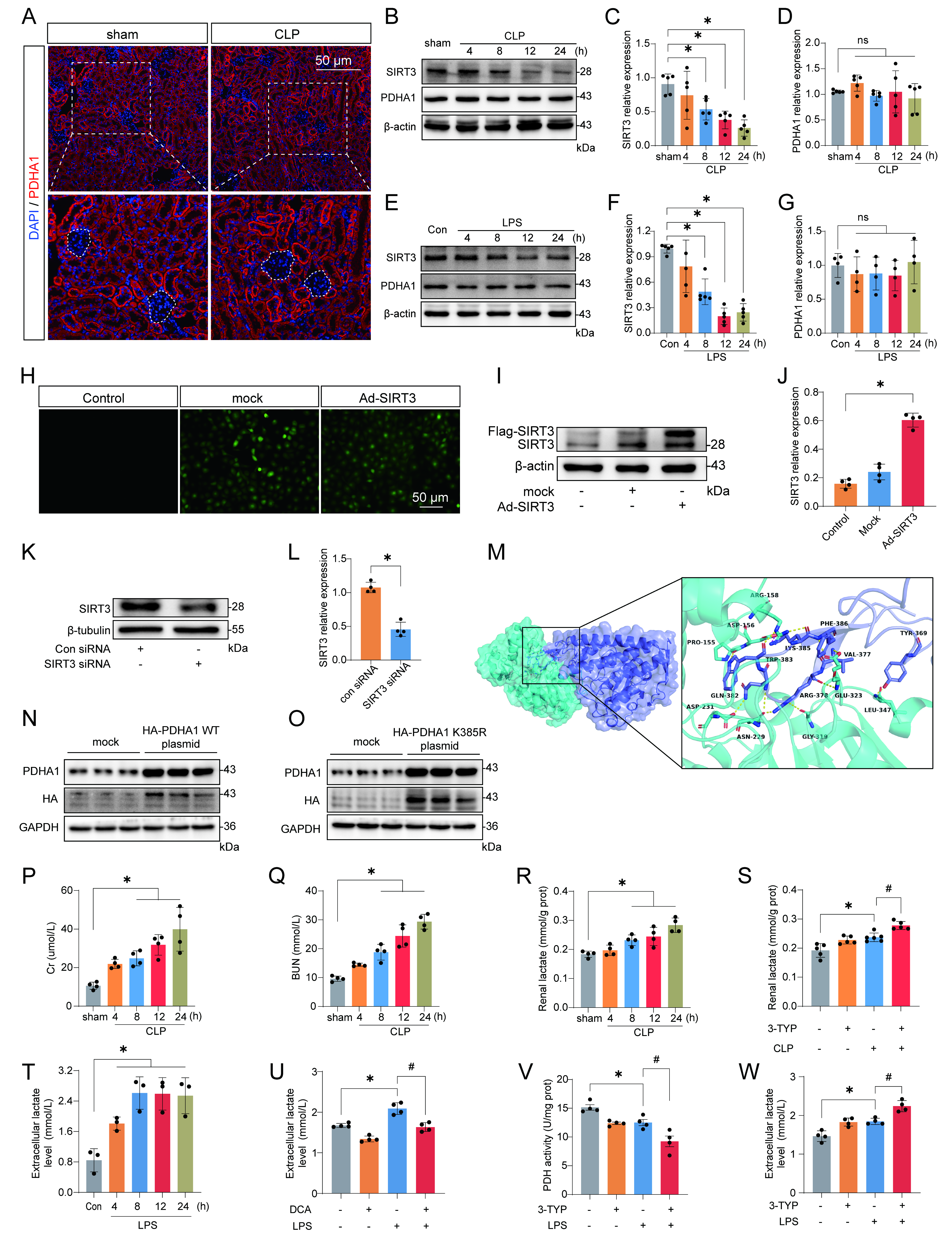

Supplement: Supplementary file 2 — Fig. S1 [file 41419_2023_5952_MOESM2_ESM.tif]

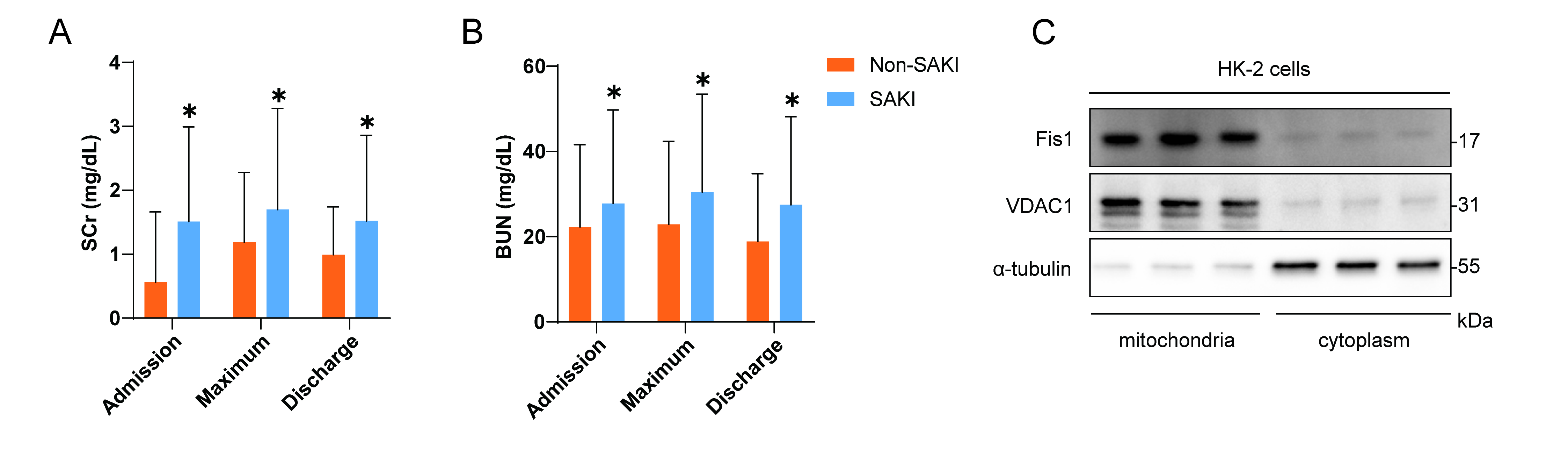

Supplement: Supplementary file 3 — Fig. S2 [file 41419_2023_5952_MOESM3_ESM.tif]

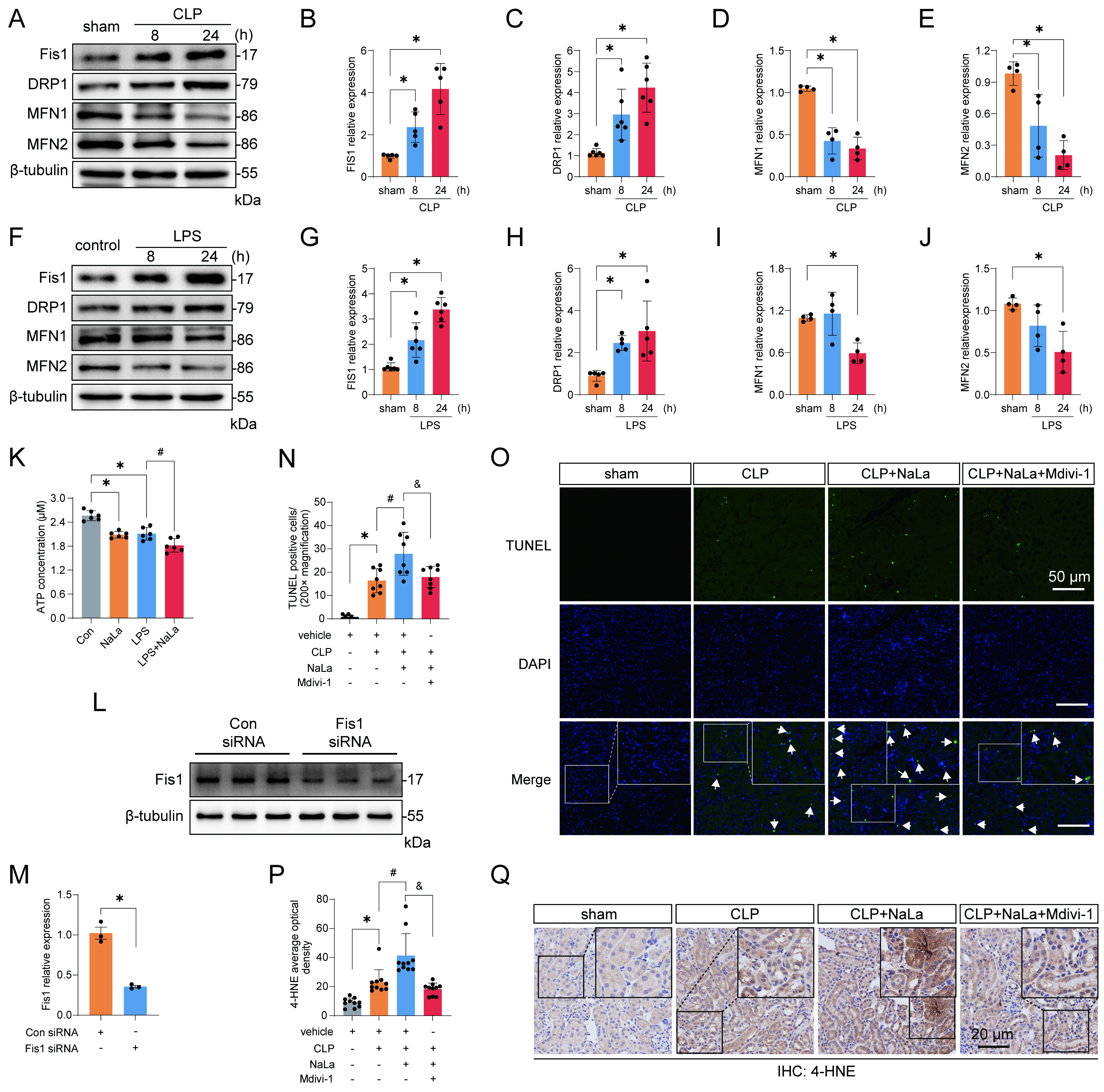

Supplement: Supplementary file 4 — Fig. S3 [file 41419_2023_5952_MOESM4_ESM.tif]

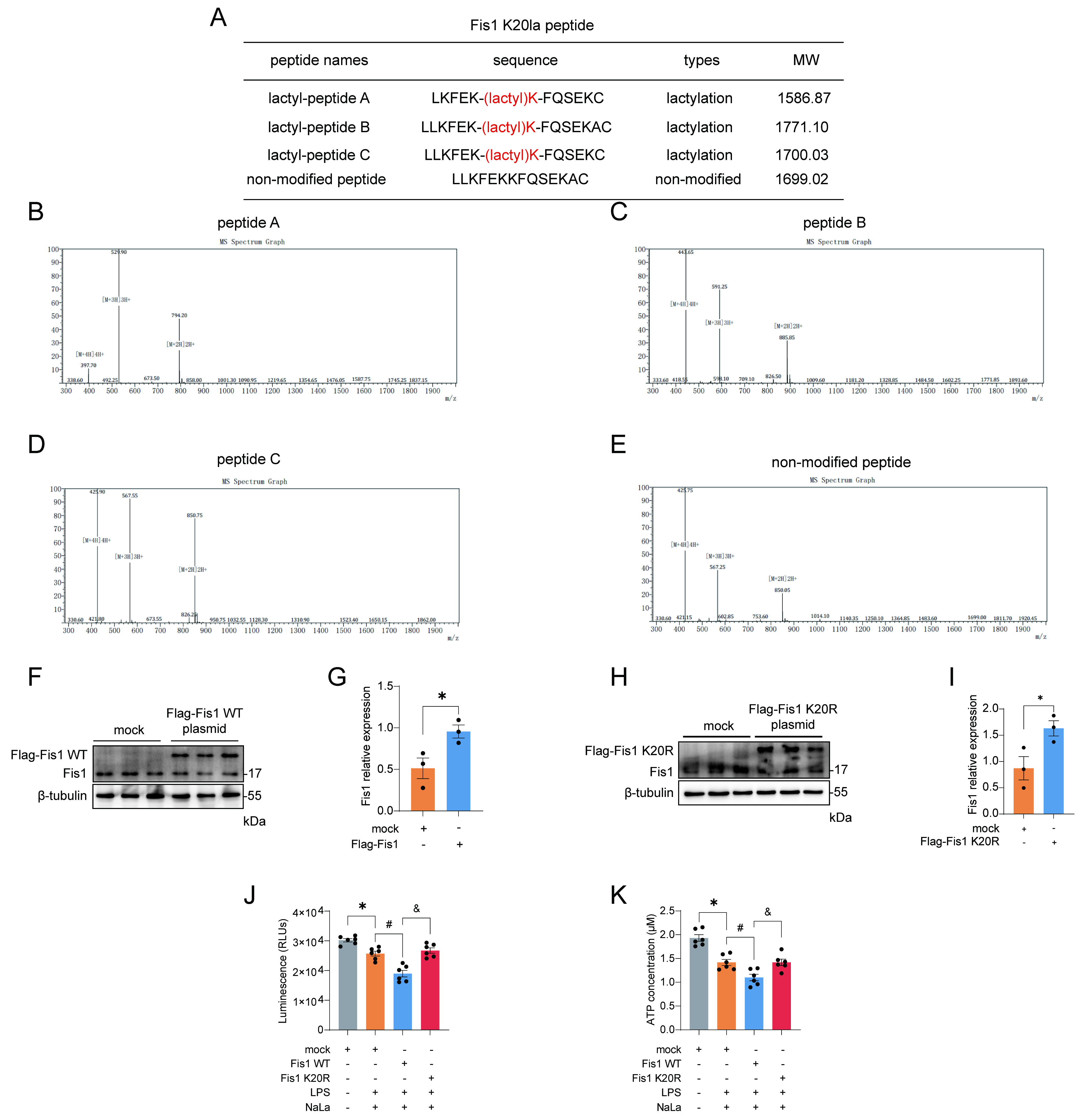

Supplement: Supplementary file 5 — Fig. S4 [file 41419_2023_5952_MOESM5_ESM.tif]

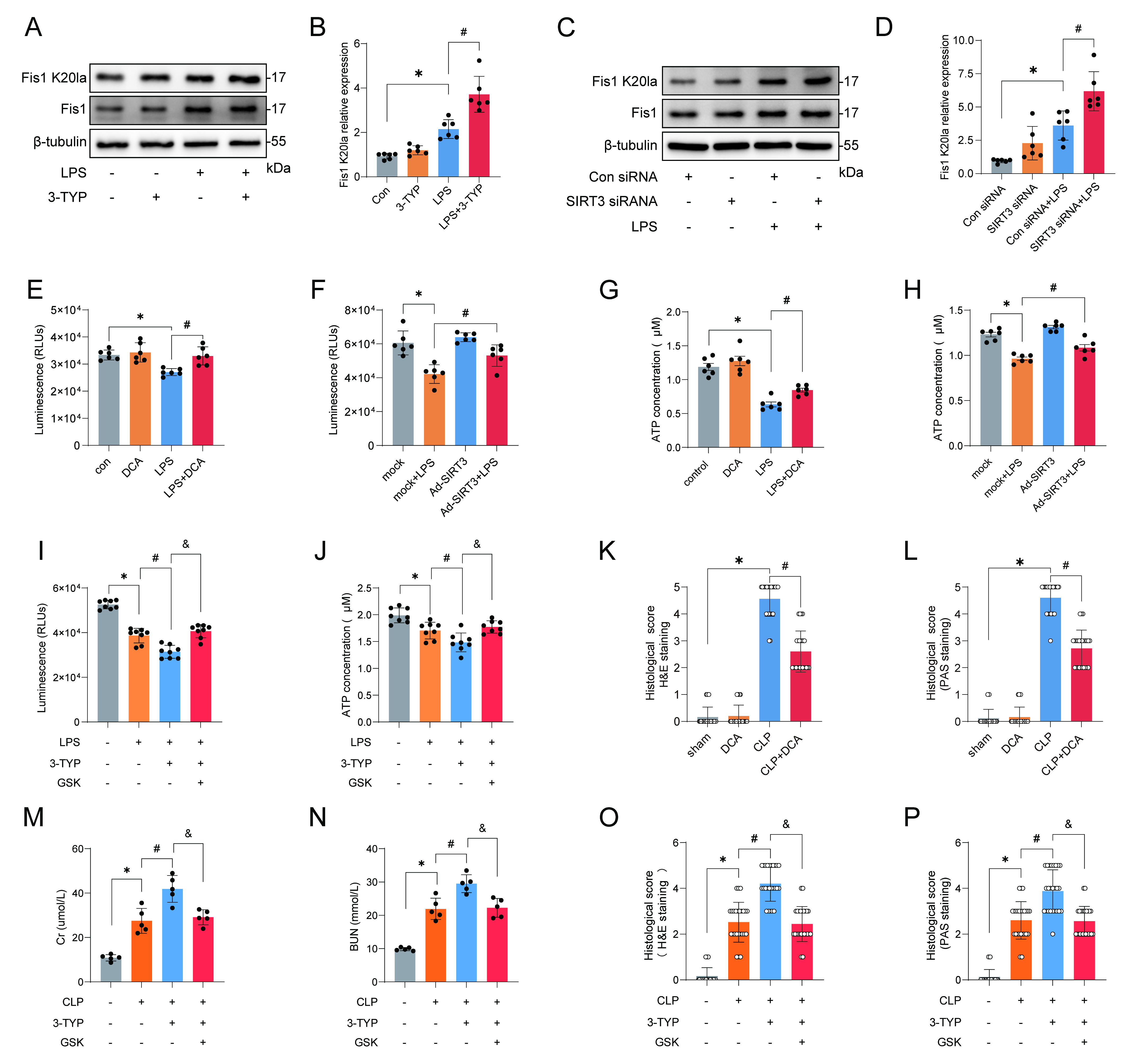

Supplement: Supplementary file 6 — Fig. S5 [file 41419_2023_5952_MOESM6_ESM.tif]

Fig. 2

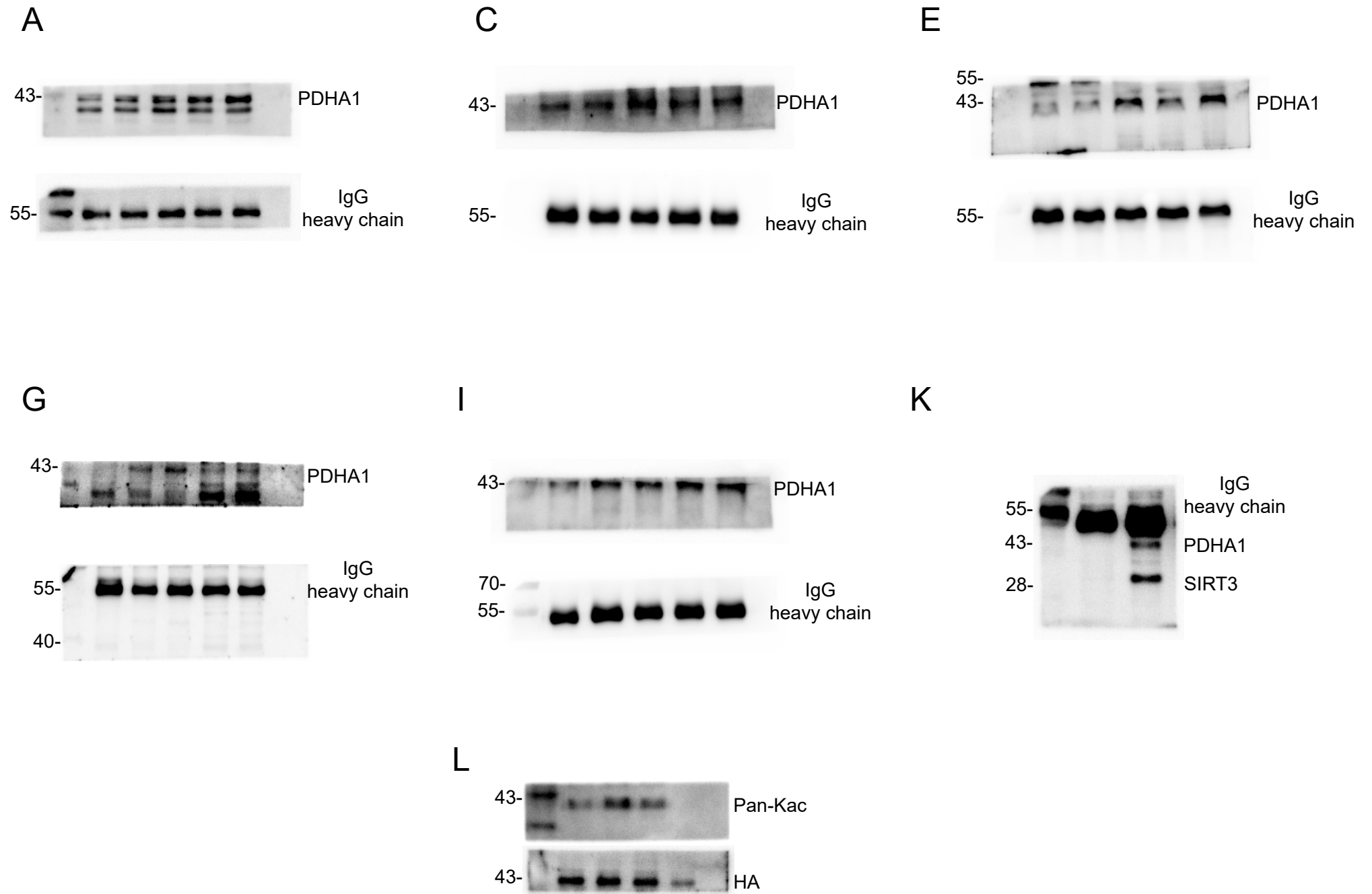

Fig. 5

A

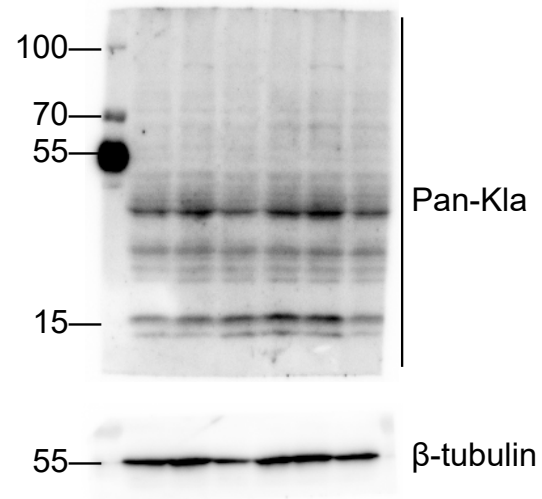

I

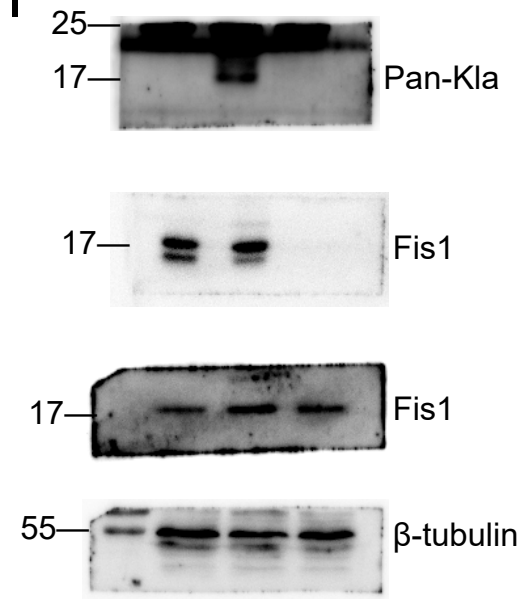

J

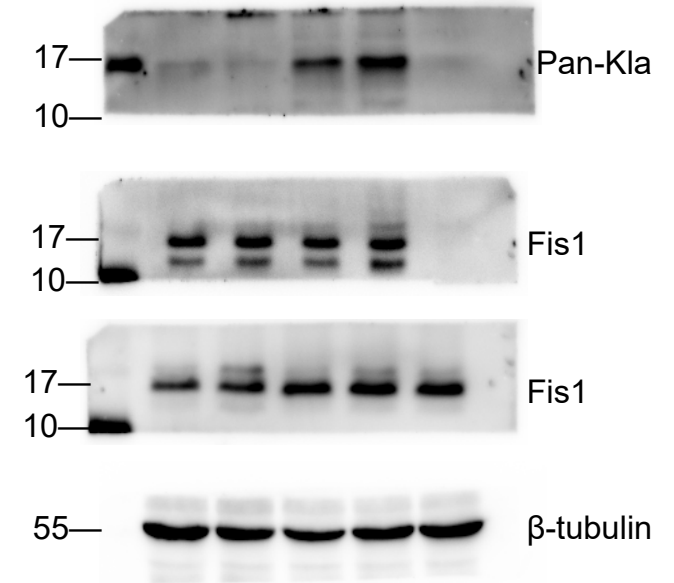

L

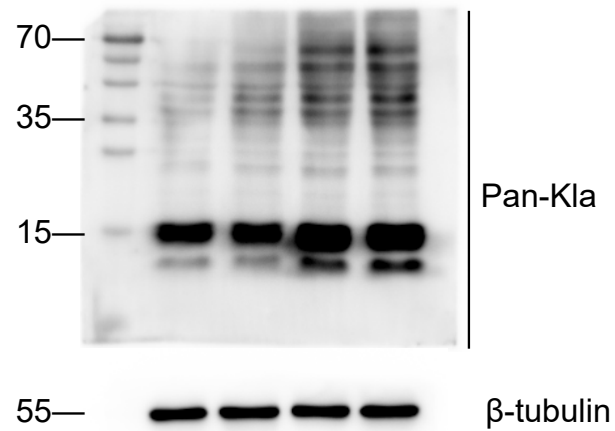

M

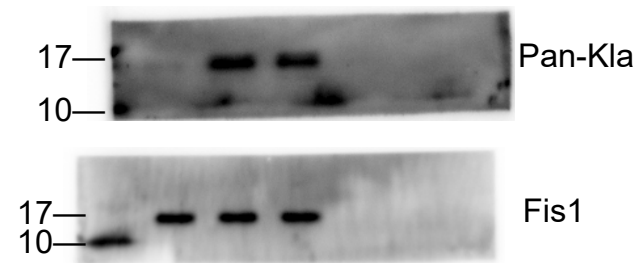

Fig. 6

A

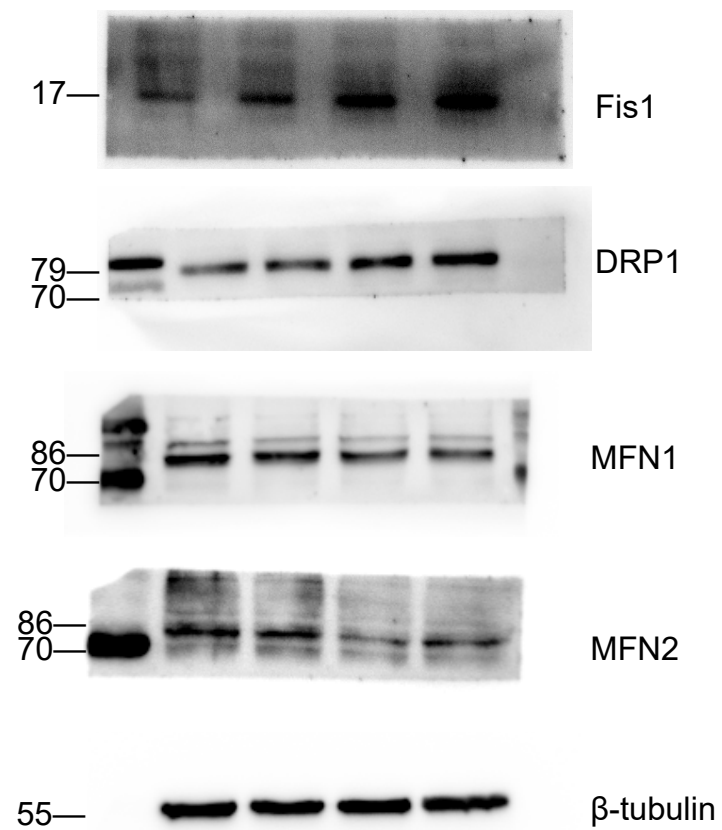

H

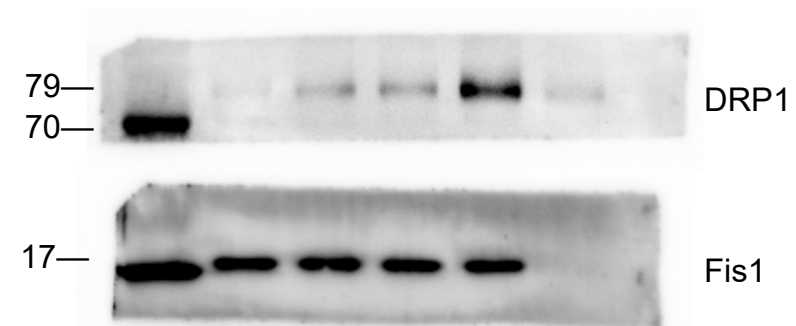

O

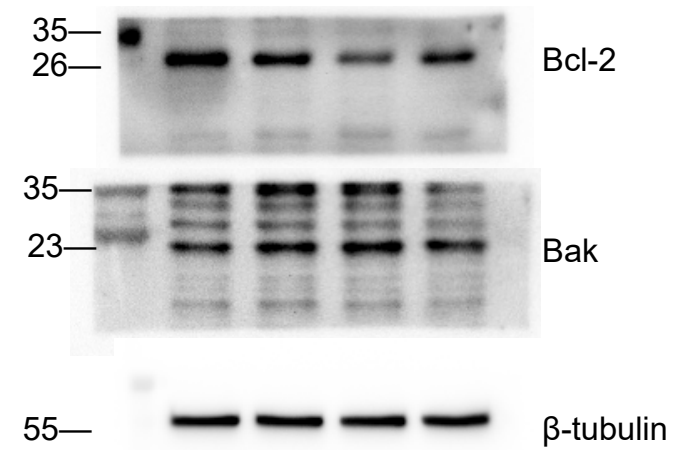

Fig. 7

B

Fis1 K20Ia

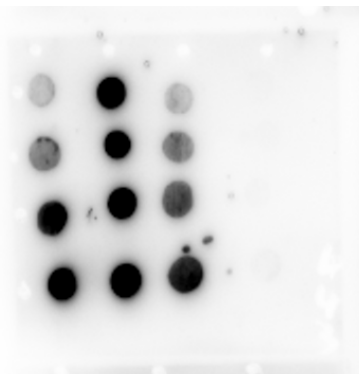

C

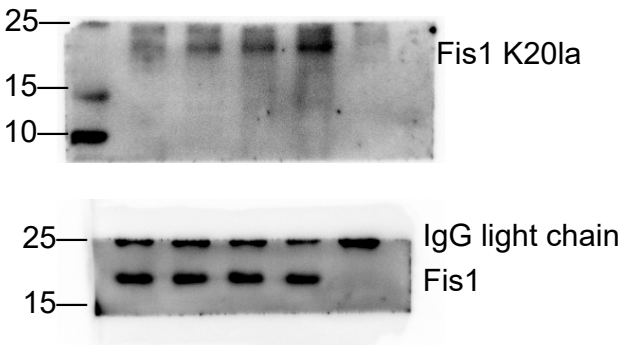

D

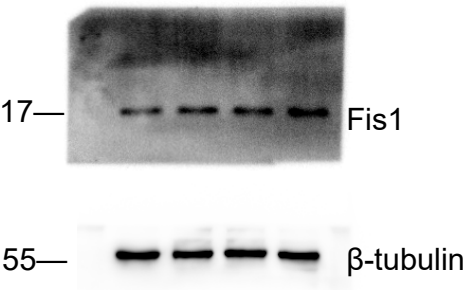

F

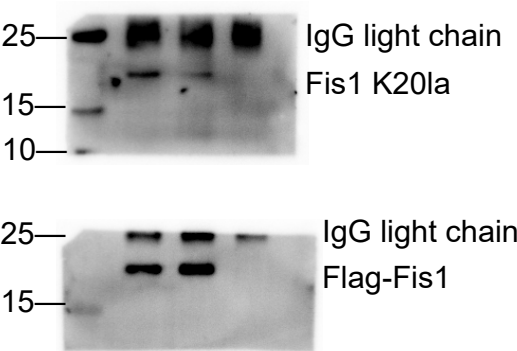

G

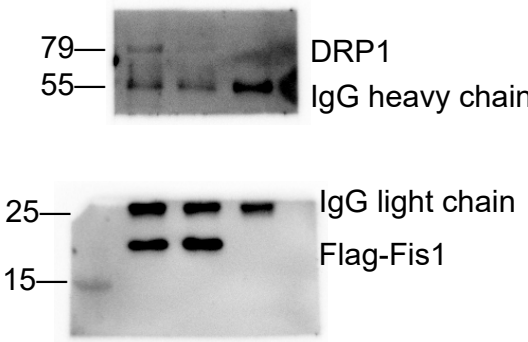

L

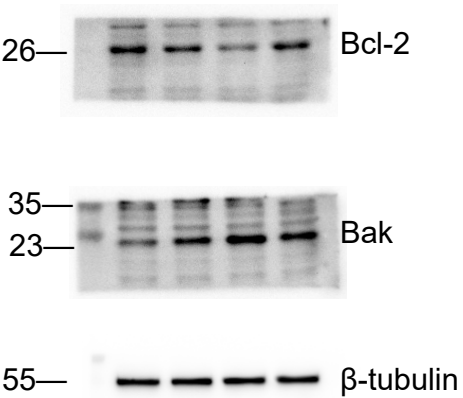

Fig. 8

A

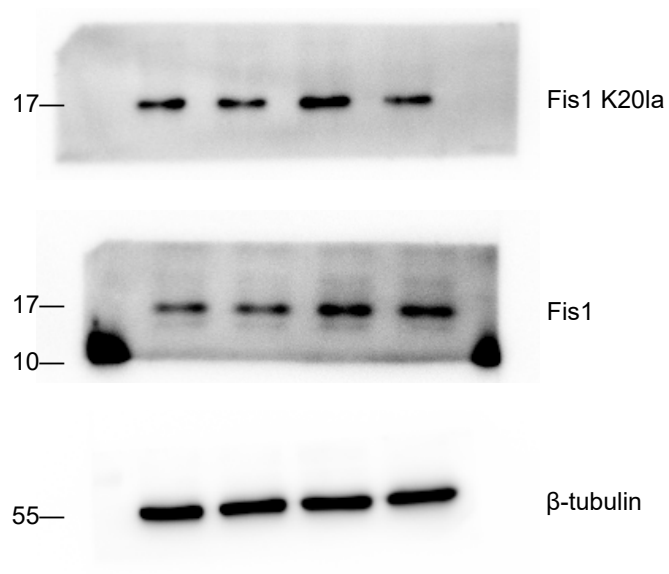

C

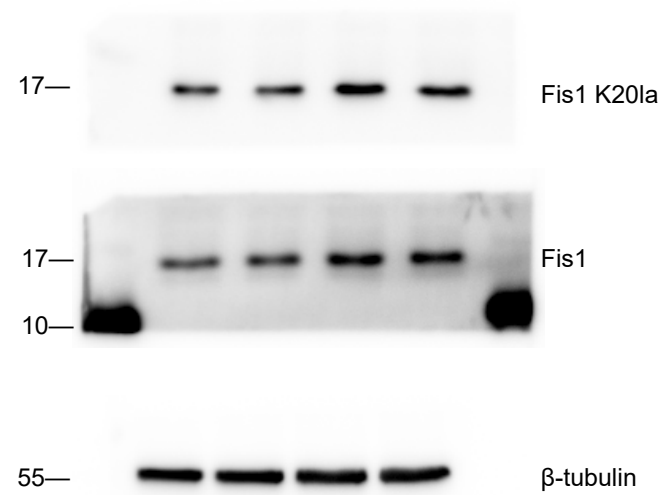

E

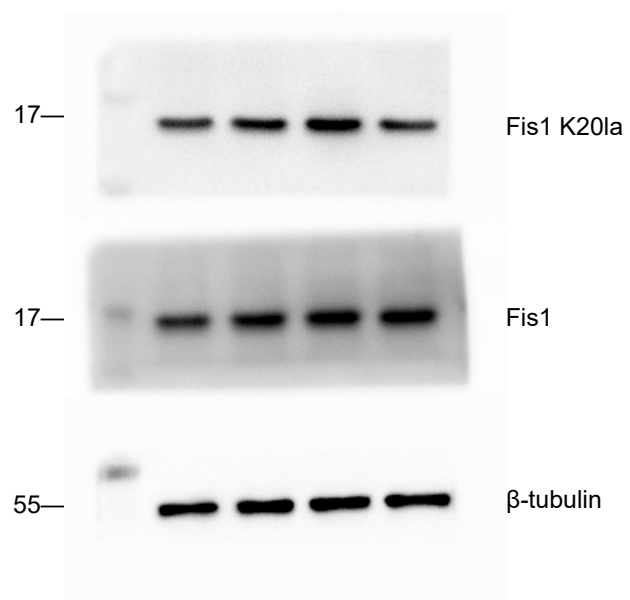

Fig. S1

B

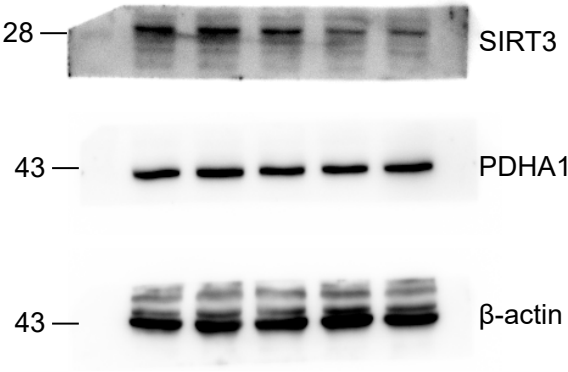

E

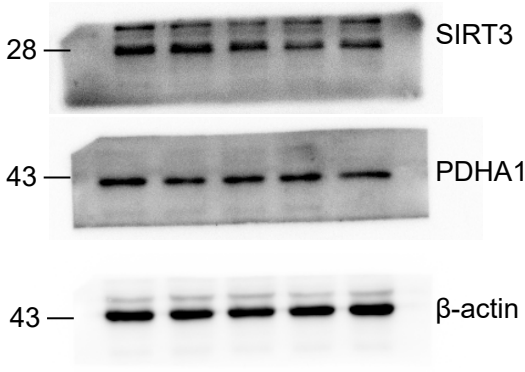

I

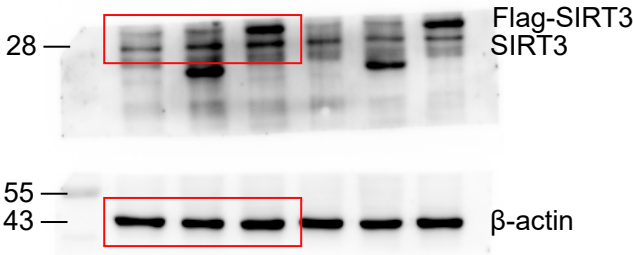

K

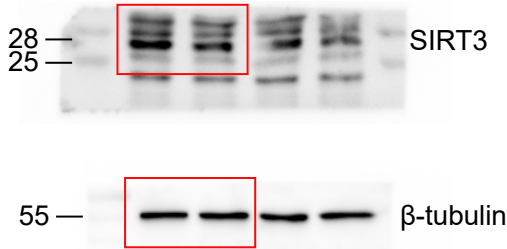

N

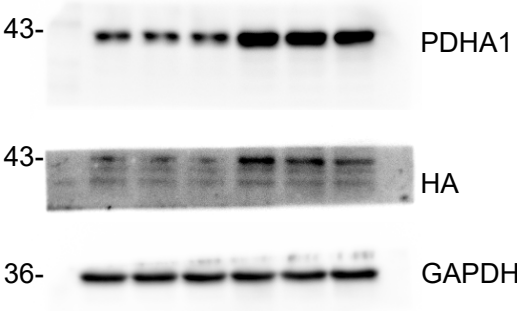

O

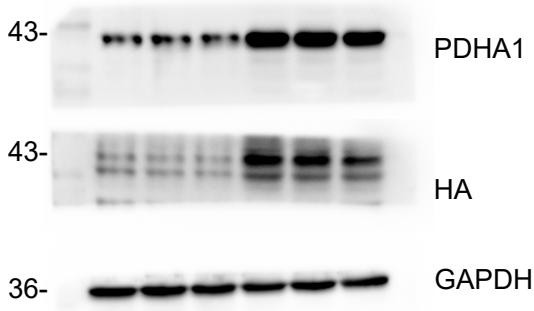

Fig. S2

C

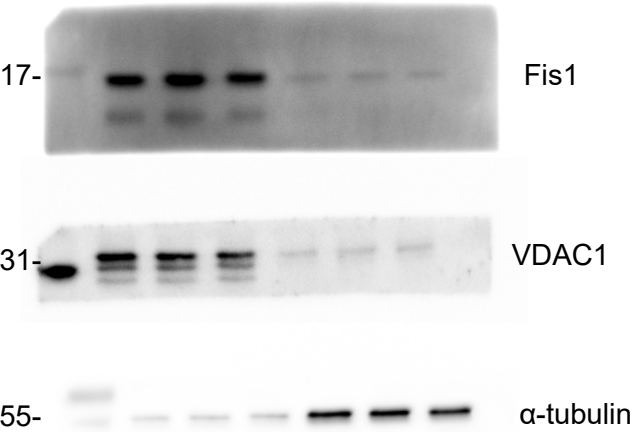

Fig. S3

A

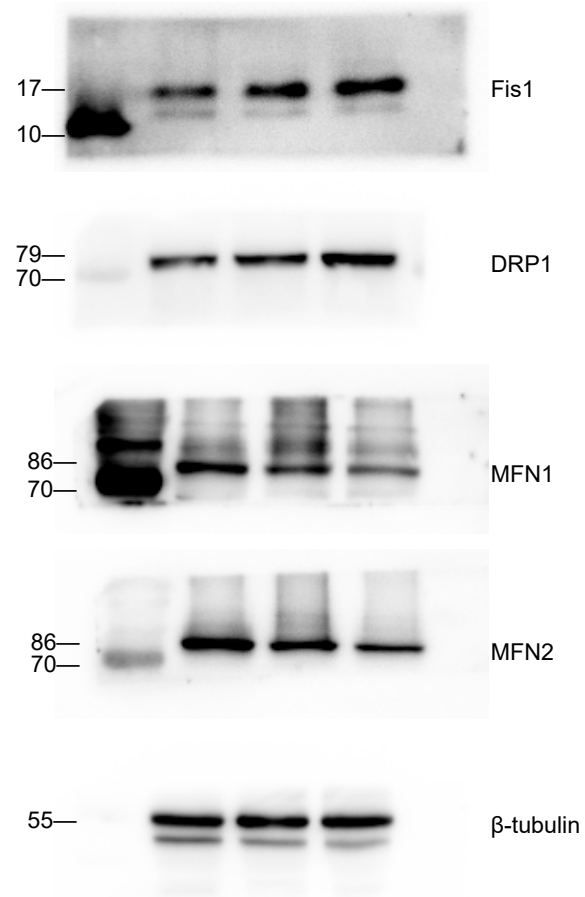

F

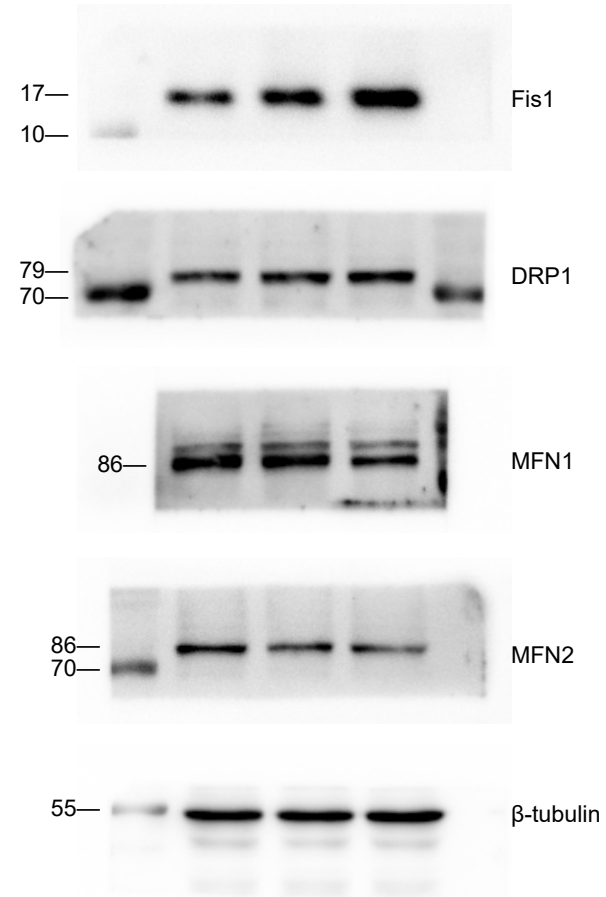

L

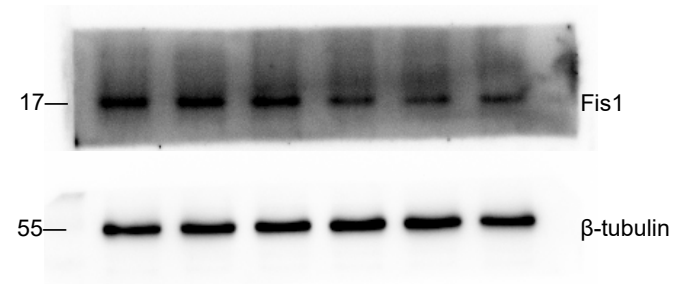

Fig. S4

F

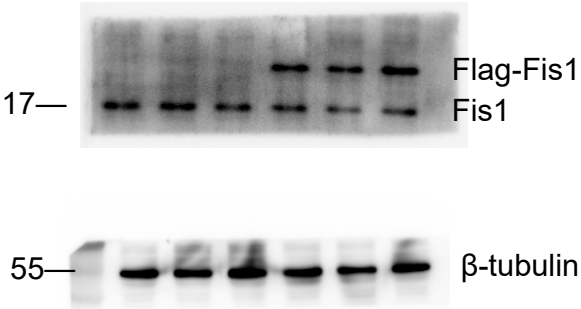

H

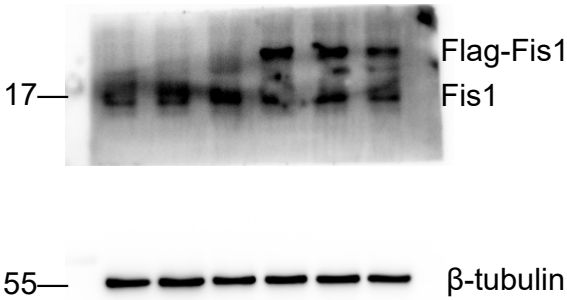

Fig. S5

A

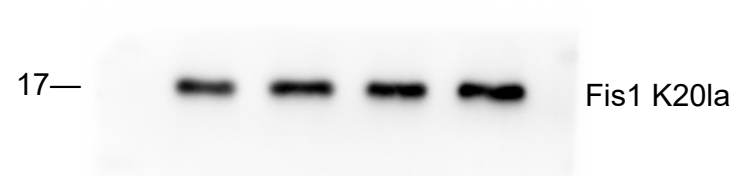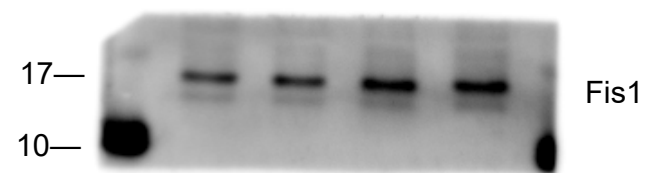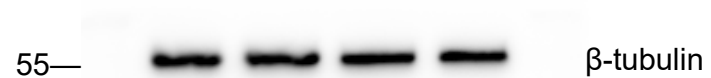

C

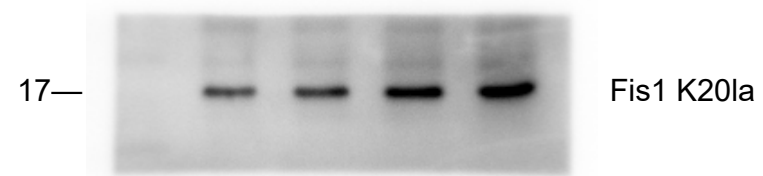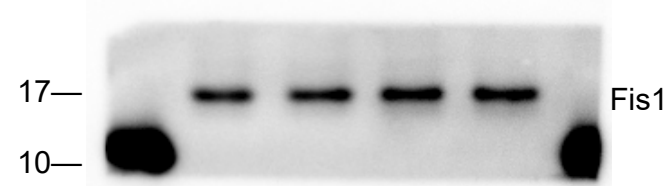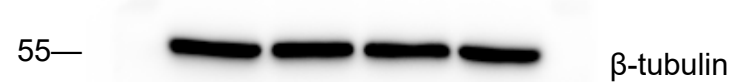

Supplement: Supplementary file 8 — Original Data File [file 41419_2023_5952_MOESM8_ESM.pdf]
