# Supplementary material for: PDHA1 hyperacetylation-mediated lactate overproduction promotes sepsis-induced acute kidney injury via Fis1 lactylation
Source: Cell Death Dis. 2023 Jul 21;14(7):457. doi: 10.1038/s41419-023-05952-4 (PMC10362039; doi:10.1038/s41419-023-05952-4)
Supplement: Supplementary file 7 — Supplementary Materials [file 41419_2023_5952_MOESM7_ESM.docx]

Supplementary Materials for

**PDHA1 hyperacetylation-mediated lactate overproduction promotes sepsis-induced acute kidney injury via Fis1 lactylation**

Sheng An, Yi Yao, Hongbin Hu, *et al.*

*Corresponding author: Zhongqing Chen, zhongqingchen2008@163.com; Zhenhua Zeng, zhenhuazeng.2008@163.com

**Table S1. Interaction residues between SIRT3 and PDHA1.**

| Receptor | Ligand | Hydrogen bond  interaction | Electrostatic interaction |
| --- | --- | --- | --- |
| PDHA1 | SIRT3 | TYR369-LEU347 | **LYS385-ASP156** |
|  |  | PHE386-GLU323 |  |
|  |  | VAL377-GLU323 |  |
|  |  | **LYS385-ARG158** |  |
|  |  | TRP383-ARG158 |  |
|  |  | TRP383-PRO155 |  |
|  |  | GLN382-ASP231 |  |
|  |  | GLN382-ASN229 |  |
|  |  | ARG378-GLU323 |  |
|  |  | ARG378-ASN229 |  |
|  |  | ARG378-GLY319 |  |

**Table S2. Characteristics of overall sepsis patients.**

|  | **Overall**  **(n=3260)** | **＜2**  **(n=1465)^*^** | **2≤;＜4**  **(n=1038)^*^** | **4≤;＜10**  **(n=627)^*^** | **≥10**  **(n=130)^*^** | ***P* value** |
| --- | --- | --- | --- | --- | --- | --- |
| Lactate(mmol/L) ^a^ | 2.2 [1.3, 3.8] | 1.30 [1.0, 1.6] | 2.7 [2.3, 3.20] | 5.6 [4.6, 7.2] | 11.9 [10.9, 14.6] | <0.001 |
| Age, year | 67.0 [55.0, 78.0] | 67.0 [54.0, 78.0] | 67.0 [56.0, 77.0] | 66.0 [53.0, 78.0] | 63.0 [52.2, 76.0] | 0.334 |
| BMI | 27.7 [23.7, 33.1] | 27.8 [23.7, 33.6] | 27.8 [23.9, 33.0] | 27.3 [23.7, 32.4] | 26.8 [23.3, 31.8] | 0.341 |
| Gender, male (%) | 1689 (51.8) | 722 (49.3) | 563 (54.2) | 328 (52.3) | 76 (58.5) | 0.036 |
| Admission type (%) |  |  |  |  |  |  |
| Elective | 519 (15.9) | 207 (14.1) | 210 (20.2) | 88 (14.0) | 14 (10.8) | <0.001 |
| Emergency | 1964 (60.2) | 895 (61.1) | 568 (54.7) | 415 (66.2) | 86 (66.2) |  |
| Urgent | 777 (23.8) | 363 (24.8) | 260 (25.0) | 124 (19.8) | 30 (23.1) |  |
| Ethnicity (%) |  |  |  |  |  |  |
| Asian | 60 (1.8) | 17 (1.2) | 17 (1.6) | 20 (3.2) | 6 (4.6) | <0.001 |
| Black | 268 (8.2) | 113 (7.7) | 82 (7.9) | 60 (9.6) | 13 (10.0) |  |
| White | 2150 (66.0) | 1013 (69.1) | 696 (67.1) | 373 (59.5) | 68 (52.3) |  |
| Other | 782 (24.0) | 322 (22.0) | 243 (23.4) | 174 (27.8) | 43 (33.1) |  |
| Co-morbidities, n(%) |  |  |  |  |  |  |
| Chronic pulmonary disease | 801 (24.6) | 384 (26.2) | 261 (25.1) | 130 (20.7) | 801 (24.6) | 0.033 |
| Congestive heart failure | 783 (24.0) | 353 (24.1) | 259 (25.0) | 143 (22.8) | 783 (24.0) | 0.696 |
| Cerebrovascular disease | 462 (14.2) | 220 (15.0) | 133 (12.8) | 95 (15.2) | 462 (14.2) | 0.244 |
| Liver disease | 339 (10.4) | 109 (7.4) | 87 (8.4) | 98 (15.6) | 339 (10.4) | <0.001 |
| Myocardial infarct | 473 (14.5) | 159 (10.9) | 174 (16.8) | 117 (18.7) | 473 (14.5) | <0.001 |
| Vital sign^a^ |  |  |  |  |  |  |
| MAP (mmHg) | 74.3 [69.1, 80.9] | 75.2 [69.7, 82.5] | 73.6 [69.1, 79.5] | 73.7 [68.3, 80.5] | 71.5 [65.0, 78.2] | <0.001 |
| Heart rate | 88.7 [77.4, 101.7] | 86.2 [75.4, 98.3] | 87.8 [77.5, 100.7] | 93.4 [81.5, 108.2] | 96.6 [84.6, 111.2] | <0.001 |
| Respiratory rate | 19.9 [17.4, 23.2] | 19.5 [17.2, 22.6] | 19.6 [17.3, 22.8] | 20.9 [18.0, 24.3] | 23.6 [20.3, 27.0] | <0.001 |
| SOFA score^b^ | 3.0 [2.0, 5.0] | 3.0 [2.0, 4.0] | 4.0 [2.0, 5.0] | 4.0 [3.0, 6.0] | 5.0 [3.0, 8.0] | <0.001 |
| Cardiovascular | 1.00 [0.0, 3.0] | 1.0 [0.0, 1.0] | 1.0 [0.0, 3.0] | 1.0 [1.0, 4.0] | 3.0 [1.0, 4.0] | <0.001 |
| Coagulation | 0.0 [0.0, 1.0] | 0.0 [0.0, 0.0] | 0.0 [0.0, 1.0] | 0.0 [0.0, 1.0] | 0.0 [0.0, 2.0] | <0.001 |
| Nervous | 0.0 [0.0, 1.0] | 0.0 [0.0, 1.0] | 0.0 [0.0, 0.0] | 0.0 [0.0, 0.0] | 0.0 [0.0, 0.0] | <0.001 |
| Liver | 0.0 [0.0, 0.0] | 0.0 [0.0, 0.0] | 0.0 [0.0, 0.0] | 0.0 [0.0, 0.0] | 0.0 [0.0, 1.0] | <0.001 |
| Renal | 0.0 [0.0, 1.0] | 0.0 [0.0, 1.0] | 0.0 [0.0, 1.0] | 0.0 [0.0, 1.0] | 1.0 [0.0, 2.0] | <0.001 |
| Respiration | 0.0 [0.0, 2.0] | 0.0 [0.0, 2.0] | 1.0 [0.0, 2.0] | 1.0 [0.0, 2.0] | 0.0 [0.0, 2.0] | 0.016 |
| Laboratory value^a^ |  |  |  |  |  |  |
| Hemoglobin (g/dL) | 9.7 [8.4, 11.3] | 9.9 [8.6, 11.4] | 9.8 [8.4, 11.4] | 9.4 [8.0, 11.2] | 8.4 [7.1, 9.7] | <0.001 |
| Platelet (×10^9^/L) | 173.0 [116.0, 241.0] | 205.0 [146.0, 281.0] | 157.0 [112.0, 219.0] | 141.0 [91.0, 203.5] | 89.0 [48.0, 158.0] | <0.001 |
| White blood cell (×10^9^/L) | 15.2 [10.7, 20.8] | 13.5 [9.9, 18.3] | 16.3 [11.5, 21.5] | 17.6 [13.1, 24.5] | 17.4 [12.1, 26.5] | <0.001 |
| Baseline Scr (mg/dl) | 0.67 (±0.25) | 0.64 (±0.24) | 0.67 (±0.24) | 0.70 (±0.25) | 0.86 (±0.24) | <0.001 |
| Arterial pH | 7.32 [7.23, 7.44] | 7.37 [7.30, 7.46] | 7.31 [7.25, 7.44] | 7.22 [7.14, 7.30] | 7.06 [6.96, 7.15] | <0.001 |
| PaO2/FiO2 ratio | 182.5 [110.0, 265.0] | 198.0 [125.0, 288.0] | 184.0 [114.3, 263.0] | 152.0 [91.0, 230.0] | 104.0 [67.3, 185.5] | <0.001 |
| Fluid balance^c^ |  |  |  |  |  |  |
| Colloid input (mL) | 63.0 [0.0, 362.2] | 0.0 [0.0, 215.0] | 102.5 [0.0, 399.0] | 204.0 [0.0, 569.0] | 500.0 [0.0, 1080.7] | <0.001 |
| Crystalloid input (ml) | 1000.0 [563.0, 1572.0] | 881.0 [500.0, 1333.0] | 1000.0 [588.0, 1750.0] | 1143.00 [744.50, 1958.00] | 1837.5 [877.5, 2850.5] | <0.001 |
| Urine output (mL) | 1527.5 [869.8, 2316.5] | 1615.0 [1020.0, 2403.0] | 1511.0 [903.2, 2238.7] | 1445.00 [597.00, 2290.00] | 337.5 [111.0, 1270.7] | <0.001 |
| Volume (mL) | -300.0 [-1216.5, 670.0] | -592.0 [-1418.0, 175.0] | -152.5 [-1039.7, 708.5] | 66.00 [-991.00, 1280.00] | 1878.0 [170.0, 3431.0] | <0.001 |
| Length of ICU stay | 6.3 [2.7, 14.2] | 6.13 [2.85, 13.99] | 5.71 [2.40, 13.33] | 8.14 [3.28, 16.28] | 4.41 [1.23, 12.49] | <0.001 |
| Length of hospital stay | 12.0 [6.6, 21.8] | 12.52 [7.04, 21.78] | 11.56 [6.58, 20.25] | 13.67 [7.00, 23.79] | 5.91 [1.40, 19.42] | <0.001 |

Abbreviations: BMI, body mass index; SOFA, sequential organ failure assessment; MAP, mean arterial pressure; SCr, serum creatinine.

* Sepsis patients was grouped by serum lactate levels, according to "< 2 mmol/L", "2≤; < 4 mmol/L", "4≤; < 10 mmol/L", "≥ 10 mmol/L".

^a^ The worst values within the first 24 h after ICU admission were recorded;

^b^ SOFA score was calculated within the first 24 h after ICU admission using the value associated with the greatest severity of illness. The neurological SOFA score in sedated patients was calculated by Glasgow coma scale (GCS) as 15 points.

^c^ The values on ICU discharge were recorded.

**Table S3. Clinical Outcomes**

| **Clinical outcomes** | **Overall (n=3260)** | **＜2**  **(n=1465)*** | **2≤;＜4**  **(n=1038)*** | **4≤;＜10**  **(n=627)*** | **≥10**  **(n=130)*** |
| --- | --- | --- | --- | --- | --- |
| AKI (%) | 2816 (86.4) | 1229 (83.9) | 895 (86.2) | 574 (91.5) | 118 (90.8) |
| Stage 1 | 443 (13.6) | 207 (14.1) | 163 (15.7) | 62 (9.9) | 11 (8.5) |
| Stage 2 | 1282 (39.3) | 603 (41.2) | 423 (40.8) | 232 (37.0) | 24 (18.5) |
| Stage 3 | 1091 (33.5) | 419 (28.6) | 309 (29.8) | 280 (44.7) | 83 (63.8) |
| RRT (%) | 312 (9.6) | 70 (4.8) | 76 (7.3) | 117 (18.7) | 49 (37.7) |
| Recovery (%) | 2082 (63.9) | 923 (63.0) | 674 (64.9) | 420 (67.0) | 65 (50.0) |
| Mortality (%) | 703 (21.6) | 199 (13.6) | 191 (18.4) | 225 (35.9) | 88 (67.7) |

Abbreviations: AKI, acute kidney injury; RRT, renal replacement therapy.

* Sepsis patients was grouped by serum lactate levels, according to "< 2 mmol/L", "2≤; < 4 mmol/L", "4≤; < 10 mmol/L", "≥ 10 mmol/L".

**Table S4 The value of BUN, SCr and urinie output of overall sepsis patients**

|  | **Overall**  **(n=3260)** | **＜2**  **(n=1465)^*^** | **2≤;＜4**  **(n=1038)^*^** | **4≤;＜10**  **(n=627)^*^** | **≥10**  **(n=130)^*^** | ***P* value** |
| --- | --- | --- | --- | --- | --- | --- |
| BUN at ICU admission (mg/dL) | 26.82 (±21.60) | 27.78 (±23.10) | 24.63 (±19.16) | 27.81 (±21.64) | 34.37 (±25.11) | <0.001 |
| BUN at ICU discharge(mg/dL) | 25.97 (±20.19) | 26.43 (±21.59) | 23.66 (±18.27) | 28.36 (±20.19) | 33.33 (±19.88) | <0.001 |
| Maximum BUN during ICU (mg/dL) | 29.15 (±22.56) | 29.71 (±24.04) | 26.54 (±20.24) | 31.47 (±22.45) | 38.89 (±24.87) | <0.001 |
| SCr at ICU admission (mg/dL) | 0.67 (±0.25) | 0.64 (±0.24) | 0.67 (±0.24) | 0.70 (±0.25) | 0.86 (±0.24) | <0.001 |
| SCr at ICU discharge (mg/dL) | 1.43 (±1.28) | 1.40 (±1.37) | 1.28 (±1.10) | 1.64 (±1.32) | 2.25 (±1.26) | <0.001 |
| Maximum SCr during ICU (mg/dL) | 1.61 (±1.51) | 1.58 (±1.61) | 1.42 (±1.23) | 1.86 (±1.57) | 2.69 (±2.10) | <0.001 |
| Urine output at ICU admission (mL) | 747.00 [330.00, 1355.00] | 780.00 [355.50, 1390.00] | 830.00 [420.00, 1410.00] | 590.00 [230.00, 1197.50] | 287.50 [35.00, 575.00] | <0.001 |
| Urine output at ICU discharge (mL) | 740.00 [358.00, 1280.00] | 800.00 [400.00, 1385.00] | 775.00 [430.00, 1255.00] | 615.00 [228.00, 1180.00] | 404.00 [175.00, 798.75] | <0.001 |
| Minimum urine output during ICU (mL) | 450.00 [200.00, 830.00] | 450.00 [200.00, 820.00] | 450.00 [200.00, 840.00] | 445.00 [190.50, 842.50] | 231.00 [66.50, 635.00] | 0.52 |
| Maximum urine output during ICU (mL) | 1125.00 [685.00, 1790.00] | 1120.00 [685.00, 1780.00] | 1145.00 [695.00, 1800.00] | 1108.00 [675.00, 1780.00] | 1132.50 [640.00, 1828.75] | 0.573 |

Abbreviations: BUN; blood urea nitrogen; SCr: serum creatinine; ICU, intensive care unit

*Sepsis patients was grouped by serum lactate levels, according to "<2 mmol/L", "2≤; <4 mmol/L", "4≤; <10 mmol/L", "≥10 mmol/L"

**Table S5 The value of BUN, SCr and urinie output between SAKI and Non-SAKI patients**

|  | **Overall (n=3260)** | **Non-SAKI (n=444)** | **SAKI (n=2816)** | ***P* value** |
| --- | --- | --- | --- | --- |
| BUN at ICU admission (mg/dL) | 26.82 (±21.60) | 22.28 (±19.30) | 27.78 (±21.93) | <0.001 |
| BUN at ICU discharge(mg/dL) | 25.97 (±20.19) | 18.86 (±15.92) | 27.47 (±20.67) | <0.001 |
| Maximum BUN during ICU (mg/dL) | 29.15 (±22.56) | 22.90 (±19.49) | 30.47 (±22.94) | <0.001 |
| SCr at ICU admission (mg/dL) | 0.67 (±0.25) | 0.56 (±1.10) | 1.51 (±1.48) | <0.001 |
| SCr at ICU discharge (mg/dL) | 1.43 (±1.28) | 0.99 (±0.75) | 1.52 (±1.34) | <0.001 |
| Maximum SCr during ICU (mg/dL) | 1.61 (±1.51) | 1.19 (±1.09) | 1.70 (±1.58) | <0.001 |
| Urine output at ICU admission (mL) | 747.00 [330.00, 1355.00] | 1165.00 [660.00, 1840.00] | 670.00 [290.50, 1226.00] | <0.001 |
| Urine output at ICU discharge (mL) | 740.00 [358.00, 1280.00] | 1160.00 [705.00, 1800.00] | 658.00 [310.00, 1153.50] | <0.001 |
| Minimum urine output during ICU (mL) | 450.00 [200.00, 830.00] | 470.00 [200.00, 855.00] | 450.00 [200.00, 825.00] | 0.165 |
| Maximum urine output during ICU (mL) | 1125.00 [685.00, 1790.00] | 1122.00 [685.00, 1750.00] | 1125.00 [685.00, 1800.00] | 0.534 |

Abbreviations: BUN: blood urea nitrogen; SCr: serum creatinine; ICU, intensive care unit.

**Supplementary materials and methods**

**Cell Culture and Treatments**

HK-2 cells are an immortalized human proximal tubular epithelial cell line derived from normal human kidneys. The cells were purchased from Kunming Cell Bank (China). HK-2 cells were cultured in medium containing 10% fetal bovine serum and DMEM/F12 in an incubator at 37 °C with 5% CO_2_ and 95% air, and the medium was changed daily. Based on previous studies of LPS usage and doses ^1^, the cells were exposed to 10 μg/ml LPS (L2880, Sigma) for a corresponding period of time for subsequent experiments. The cells were pretreated with 5 mM DCA, 50 μM 3-TYP, 10 mM NaLa, 10 μM Mdivi-1 and 5 μM GSK for 0.5 h before LPS stimulation.

**The TMT-labelled Acetylome and 4D Label-free Lactylome**

Mitochondria were extracted from the kidney cortices of mice in the sham group and CLP group using a Tissue Mitochondrial Isolation Kit (C3606, Beyotime). The extracted mitochondria were used for the TMT-labelled acetylome. For the 4D label-free lactylome, whole protein from the renal cortices of mice in sham group and CLP group was used. Acetylome and lactylome analyses were performed by PTM Bio (China). The screening of the differentially modified protein sites followed the following criteria: 1.5 or 1/1.5 times change threshold; t test *P* < 0.05.

**siRNA, Plasmid, and Adenovirus Transfection**

The methods for siRNA, plasmid, and adenovirus transfection were described in our previous study ^2, 3^. The sequences of the oligonucleotides for the control, SIRT3 and Fis1 siRNA were synthesized by GenePharma (China). The siRNA-targeted sequences were as follows: control siRNA (sense 5'-UUCUCCGAACGUGUCACGUTT-3' and antisense 5'-ACGUGACACGUUCGGAGAATT-3'), SIRT3 siRNA (sense 5'-GGUGGAAGAAGGUCCAUAUTT-3' and antisense 5'-AUAUGGACCUUCUUCCACCTT-3'), and Fis1 siRNA (sense 5'-GCAAGUACAAUGAUGACAUCCGUAA-3’ and antisense 5’-UUACGGAUGUCAUCAUUGUACUUGC-3’). The adenoviral vectors (expressing GFP- and flag-tagged SIRT3 (Ad-SIRT3) and empty vector) and plasmids (HA-PDHA1 wild type, HA-PDHA1 K385R, Flag-Fis1 wild type, Flag-Fis1 K20R, and empty plasmid) were synthesized by Genechem (China).

**Western Blotting**

Renal tissues and HK-2 cells were lysed in RIPA Lysis Buffer (P0013B, Beyotime). Quantification of the Western blots was performed by ImageJ.

The antibodies used were as follows: anti-SIRT3 (1:1000; 5490S, CST), anti-DRP1 (1:1000; 8570S, CST), anti-PDHA1 (1:5000; 18068-1-AP, Proteintech), anti-Fis1 (1:1000; 10956-1-AP, Proteintech), anti-Bcl-2 (1:2000; 12789-1-AP, Proteintech), anti-Bak (1:2000; 29552-1-AP, Proteintech), anti-MFN1 (1:1000;13798-1-AP, Proteintech), anti-MFN2 (1:2000; 12186-1-AP, Proteintech), Pan Lactyl-Lysine (1:1000; PTM-1401, PTM Bio), anti-Fis1 K20la (1:1000, PTM Bio), Anti-β-actin (1:5000; RM2001, Beijing Ray Antibody Biotech), anti-β-tubulin (1:5000; RM2003, Beijing Ray Antibody Biotech), anti-α-tubulin (1:5000; RM2007, Beijing Ray Antibody Biotech), anti-VDAC1 (1:1000; RM2010, Beijing Ray Antibody Biotech), anti-HA tag (1:5000; 51064-2-AP, Proteintech), anti-Flag tag (1:5000; RM1002, Beijing Ray Antibody Biotech), anti-Mouse IgG(H+L)-HRP (1:5000; RM3001, Beijing Ray Antibody Biotech), and anti-Rabbit IgG(H+L)-HRP (1:6000; RM3002, Beijing Ray Antibody Biotech).

**Immunoprecipitation**

Renal tissues and HK-2 cells were lysed in IP Lysis Buffer (P0013, Beyotime). Protein A+G Magnetic Beads (P2108, Beyotime) were used for IP according to the manufacturer’s instructions. The antibodies used were pan Acetyl-Lysine (1:100; A2391, Abconal), rabbit IgG (1:200; B900610, Proteintech), anti-Fis1 (4 μg; 10956-1-AP, Proteintech), and anti-Flag tag (1:100; RM1002, Beijing Ray Antibody Biotech), anti-HA tag (4 μg; 51064-2-AP, Proteintech).

**PDH Activity Assay**

PDH activity was determined by a PDH Activity Assay Kit (BC0385, Solarbio) according to the manufacturer's instructions. PDH catalyzes the dehydrogenation of pyruvate with concomitant reduction of 2, 6-dichlorophenol-indophenol (2, 6-DCPIP), resulting in a decrease in light absorption at 605 nm. The consumption of 1 nmol of 2, 6-DCPIP per minute in the reaction system per mg protein is defined as one unit of enzyme activity (U/mg prot).

**Lactate, ATP Production and Cell Viability Assay**

Lactate and ATP levels were assessed using a Lactate Assay Kit (A019-2-1, Nanjing Jiancheng Bioengineering Institute) and Enhanced ATP Assay Kit (S0027, Beyotime) respectively, according to the manufacturer's instructions. Cell viability was measured using a CellTiter-LumiTM Plus Luminescent Cell Viability Assay Kit (C0068M, Beyotime).

**Molecular Docking**

The 3D structure of SIRT3 (UniProt ID: Q9NTG7) and PDHA1(UniProt ID: P08559) were obtained from Protein Data Bank (SIRT3 ID: 3GLR, PDHA1 ID: 2OZL). Molecular modeling was performed in AutoDockTools-1.5.7 and ZDock as described previously ^4, 5, 6^, followed by visualizing in PyMol (Schrodinger, LLC, USA).

**Retrospective Cohort Study of the MIMIC-IV Database**

Population: Patients with sepsis within 48 h of ICU admission were eligible for inclusion in the study. Sepsis was defined according to the Sepsis 3.0 criteria ^7^. If patients were admitted more than once, only their first stay was included in the analysis. Patients were excluded from the study if they were younger than 18 years, were discharged or died within 24 h after ICU admission, had chronic kidney disease (CKD), and had a disease or took a drug that affects lactate metabolism ^8^, such as those with diabetes, malignancy, HIV infection, mitochondrial encephalomyopathy or severe liver disease at admission, those who had ever received metformin, linezolid or antiretroviral therapy, or if the potential risk variables for death were missing more than 5% ^9^.

Data collection and definitions: The data were extracted from a large, online, open free critical care database, the Multiparameter Intelligent Monitoring in Intensive Care Database IV (MIMIC-IV) ^10^, which contains ICU patient data from the Beth Israel Deaconess Medical Center between 2008 and 2019. This database was approved by the Institutional Review Board (IRB) of the Massachusetts Institute of Technology (MIT). One author (Hongbin Hu) in this study obtained access and was responsible for the data extraction (certification no. 36220489). Data on the first day of ICU admission and discharge were extracted from the MIMIC-IV database, including age, gender, ethnicity, admission type, comorbidities, SOFA score, laboratory values, fluid balance, RRT, length of hospital stay and mortality. Patients with AKI were identified and classified on the basis of the highest serum Cr (SCr) level and urine output according to the Kidney Disease Improving Global Outcomes (KDIGO) ^11^. The minimum SCr within the 7 days before admission was used as the baseline SCr. When the preadmission SCr was not available, the first SCr measured at admission was used as the baseline ^12^. AKI stages were defined by both SCr and the volume of urine output during the first 48 h after ICU admission ^11^.

Outcomes: The occurrence and severity of renal function was the primary endpoint. The use of RRT, recovery of renal function and in-hospital mortality were considered secondary outcomes. Recovery of kidney function was defined as discharge from the ICU with SCr less than 1.5 times the baseline value and normal urine output (>0.5 mL/kg/h for 24 h on discharge) ^9^.

**Mitochondrion Extraction**

The cytoplasm and mitochondria of HK-2 cells were extracted by Mitochondrion Extraction Kit (KTP4003, Abbkine), according to the manufacturer's instructions.

**Mitochondrial Morphology Assessment**

Cells were washed twice with PBS and stained with 200 nM MitoTracker™ Red CMXRos (M7512, Invitrogen) for 25 min in the dark. After being stained, the cells were washed three times with PBS, and then fixed with 4% paraformaldehyde for 15 min. After bening fixed, the cells were washed three times with PBS, and the mitochondrial morphology was assessed by confocal microscopy. Images were quantitated and analyzed by ImageJ software.

**Measurements of Mitochondrial ROS**

The generation of mitochondrial ROS was detected by the MitoSOX™ Red Mitochondrial Superoxide Indicator (M36008, Invitrogen). After being digested with trypsin, the cells were washed twice with PBS and stained with 2.5 μM MitoSox for 30 min. Then the cells were washed twice with PBS and analyzed by flow cytometry.

**TUNEL Assay, Immunohistochemistry, and Immunoﬂuorescence Staining**

Renal cell apoptosis was assessed by terminal deoxynucleotidyl transferase dUTP mediated nick-end labeling (TUNEL) assay, as described in our previously reported study ^3^.

Immunohistochemistry of Pan-Kla was performed in mouse kidney paraffin sections. The antibodies used were as follows: Pan Lactyl-Lysine (1:1600, PTM-1401RM, PTM BIO), Goat anti-rabbit-HRP (1:2000, ab205718, Abcam). Immunofluorescence staining of PDHA1, Fis1, Pan-Kla, and TOM20 were performed in mouse kidney paraffin sections. The antibodies used were as follows: anti-PDHA1 (1:200, 18068-1-AP, Proteintech), anti-Fis1 (1:100, 10956-1-AP, Proteintech), Pan Lactyl-Lysine (1:200, PTM-1401RM, PTM BIO), anti-TOM20 (1:400, 42406S, CST), Goat Anti-Rabbit IgG (HRP) (1:4000, ab205718, Abcam), Alexa Fluor®594 donkey anti-rabbit lgG(H+L) (1:400, A21207, Life Technologies), CY5 (1:400, 11066, Aatbio), and FITC (1:300, 11060, Aatbio). Nuclei were stained with DAPI (1:500). Fluorescence signals were visualized under Fluorescence Microscope (Olympus BX53).

**Histopathological Damage Score**

The score of HE staining and PAS staining were described in our previous study ^3^. Briefly, the scoring principle was as follows: 0 points for no damage; 1 point for damage less than 10%; 2 points for damage between 11-25%; 3 points for damage between 26-45%; and 4 points for damage between 46-75%; and 5 points for damage greater than 76%.

**Statistical Analysis**

Basic research data are expressed as the mean±standard deviation (mean±SD), and GraphPad Prism 9.3.0 software was used for statistical analysis. The independent sample t test was used for comparisons between two groups. One-way analysis of variance (ANOVA) and Tukey’s multiple-comparison test were used to compare data among more than two groups. The log-rank test was used for survival analysis. *P*< 0.05 was a significant difference.

In the cohort study, continuous variables are expressed as the median [IQR], and differences between groups were identified using the Kruskal-Wails H test because of their nonnormal distribution. Categorical variables are expressed as numbers and percentages, and comparisons between groups were made using the chi-square test or Fisher’s exact test. The effect of serum lactate concentrations on the occurrence of SAKI and recovery of renal function, as well as RRT requirements, was estimated using a logistic regression model. A Cox regression model was used to estimate the relationships between lactate and mortality outcomes, adjusting for confounding variables selected based on a *P* < 0.05 in univariate analysis, and potential confounders judged by the clinical expertise of our team. The relationship between the serum lactate concentrations and outcomes was also evaluated in restricted cubic spline. All statistical analyses were performed using R version 3.6.3.

**References:**

1. Wei S, Gao Y, Dai X, Fu W, Cai S, Fang H*, et al.* SIRT1-mediated HMGB1 deacetylation suppresses sepsis-associated acute kidney injury. *Am J Physiol Renal Physiol* 2019, **316**(1)**:** F20-f31.

2. Wu J, Deng Z, Sun M, Zhang W, Yang Y, Zeng Z*, et al.* Polydatin protects against lipopolysaccharide-induced endothelial barrier disruption via SIRT3 activation. *Lab Invest* 2020, **100**(4)**:** 643-656.

3. Sun M, Li J, Mao L, Wu J, Deng Z, He M*, et al.* p53 Deacetylation Alleviates Sepsis-Induced Acute Kidney Injury by Promoting Autophagy. *Front Immunol* 2021, **12:** 685523.

4. Morris GM, Huey R, Olson AJ. Using AutoDock for ligand-receptor docking. *Current protocols in bioinformatics* 2008, **Chapter 8:** Unit 8.14.

5. Pierce BG, Wiehe K, Hwang H, Kim BH, Vreven T, Weng Z. ZDOCK server: interactive docking prediction of protein-protein complexes and symmetric multimers. *Bioinformatics (Oxford, England)* 2014, **30**(12)**:** 1771-1773.

6. Pierce BG, Hourai Y, Weng Z. Accelerating protein docking in ZDOCK using an advanced 3D convolution library. *PloS one* 2011, **6**(9)**:** e24657.

7. Singer M, Deutschman CS, Seymour CW, Shankar-Hari M, Annane D, Bauer M*, et al.* The Third International Consensus Definitions for Sepsis and Septic Shock (Sepsis-3). *JAMA* 2016, **315**(8)**:** 801-810.

8. Kraut JA, Madias NE. Lactic acidosis. *N Engl J Med* 2014, **371**(24)**:** 2309-2319.

9. Zhao GJ, Xu C, Ying JC, Lü WB, Hong GL, Li MF*, et al.* Association between furosemide administration and outcomes in critically ill patients with acute kidney injury. *Crit Care* 2020, **24**(1)**:** 75.

10. Johnson A, Bulgarelli, L., Pollard, T., Horng, S., Celi, L. A., & Mark, R. MIMIC-IV (version 1.0). 2021 [cited]Available from: <https://physionet.org/content/mimiciv/1.0/>

11. Khwaja A. KDIGO clinical practice guidelines for acute kidney injury. *Nephron Clinical practice* 2012, **120**(4)**:** c179-184.

12. De Rosa S, Samoni S, Ronco C. Creatinine-based definitions: from baseline creatinine to serum creatinine adjustment in intensive care. *Crit Care* 2016, **20:** 69.
